# Electrospun CoFe_2_O_4_ Nanowires Tailored for Magnetoelectrochemistry

**DOI:** 10.1021/acsnano.5c03628

**Published:** 2025-08-06

**Authors:** Zexuan Wang, María F. Navarro Poupard, Ramsundar Rani Mohan, Loukya Boddapati, Jijun Zhang, Saeed Kamali, Chiara Biz, Mauro Fianchini, Francis Leonard Deepak, Jose Gracia, Laura M. Salonen, Yury V. Kolen’ko

**Affiliations:** † 246702 International Iberian Nanotechnology Laboratory, Braga 4715-330, Portugal; ‡ Department of Physics and Astronomy, 5235 Middle Tennessee State University, Murfreesboro, Tennessee 37132, United States; § Department of Mechanical, Aerospace and Biomedical Engineering, University of Tennessee Space Institute, Tullahoma, Tennessee 37388, United States; ⊥ MagnetoCat SL, Calle General Polavieja 9, 3Izq, Alicante 03012, Spain; ∥ Departamento de Química Física, Universidad de Alicante, Carretera San Vicente del Raspeig, 03690 San Vicente del Raspeig, 03080 Alicante, Spain; # 16784 CINBIO, Universidade de Vigo, Department of Organic Chemistry, 36310 Vigo, Spain

**Keywords:** magnetic nanofibers, microstructure, magnetic catalysts, oxygen evolution reaction, spin polarization, nanotechnology

## Abstract

In view of a future green hydrogen economy, the development of efficient, platinum-group-metal-free catalysts for the oxygen evolution reaction (OER) remains an important goal. Magnetic enhancement of oxygen-evolving catalysis is an interesting strategy to boost catalyst activity, as it can promote the formation of triplet oxygen through spin polarization. Magnetoelectrochemistry can rely on the use of an external magnetic field or the internal magnetic order of the catalyst. Therefore, synthesis strategies that allow for tailoring the magnetic properties of magnetic catalyst materials are of high interest. Here, we report on the synthesis of CoFe_2_O_4_ (CFO) nanowires through an electrospinning template strategy followed by calcination. The calcination temperature was found to have a profound impact on both the morphology and the magnetic properties of the materials, with a temperature of 1173 K yielding intergrown nanoparticles that formed a nanowire-like structure with excellent magnetic properties: a high saturation magnetization of 88.9 emu/g and a coercivity of 17 100 Oe at 2 K. Electron microscopy was employed to identify the temperature-dependent evolution of the microstructure of the synthesized CoFe_2_O_4_ anisotropic structures. Thereafter, the sample was studied as a catalyst for electrochemical OER in alkaline electrolyte, where its great performance was found to be further boosted by application of an external magnetic field of 500 mT, resulting in an enhancement by over 100% at a constant potential of 1.60 V_RHE_, placing CFO–1173 K among the best-performing catalyst materials in terms of magnetocurrent.

## Graphical abstract

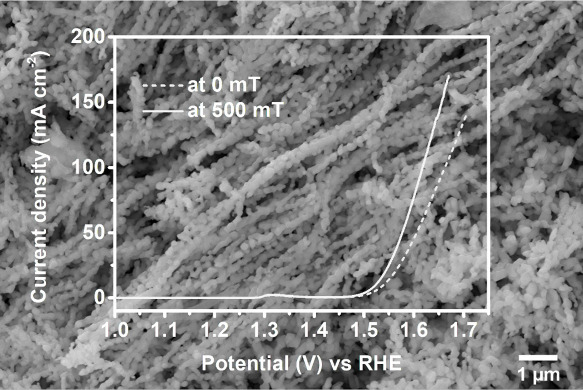

## Introduction

Green hydrogen is foreseen to play a key role as a clean energy source in the circular economy of the future,^1^ and water electrolysis, 2H_2_O (*l*) → 2H_2_ (*g*) + O_2_ (*g*), powered by renewable energy is the leading contender for its production. The kinetically slow anodic oxygen evolution reaction (OER), due to multielectron transfer, still remains a great challenge, resulting in a large overpotential for this half-reaction.^2^ Therefore, the development of highly active OER catalysts is of extreme importance.

Platinum-group metal (PGM) catalysts are prevalent under the best-performing catalyst materials for water electrolysis due to their high activity and stability under typically harsh acidic or alkaline conditions. Their scarcity and high price, however, have directed intense research efforts toward their reduction and replacement. To this end, strategies such as alloying, employing economical supports, and nanostructuring have been shown to allow for the reduction of PGMs while retaining their high activity.^3^

On the other hand, magnetic enhancement of oxygen-evolving electrocatalysis has recently received increasing attention in the literature.^4^ This is related to the influence of magnetic order on the spin selectivity of the catalytic process: oxygen has a triplet ground state, ^3^O_2_, with parallel spin alignment, whereas water and OH^–^ have antiparallel spins. Spin polarization can, therefore, increase the efficiency of the OER by promoting the generation of triplet oxygen.

Interestingly, magnetic order can be exploited to boost catalytic activity through an external magnetic field or by tailoring the internal magnetic order of the catalyst. Application of an external magnetic field promotes the alignment of the internal magnetic domains of the catalyst^5^ and was shown by Galán-Mascarós and co-workers^6^ to result in a decrease in the overpotential required to reach a current density of 100 mA/cm^2^ by ∼15 mV in alkaline OER, with the best results obtained with highly magnetic Ni–Fe oxides. Later, using Ni_4_FeO_*x*_, they demonstrated the external magnetic field to accelerate the reaction kinetics and change the reaction order, possibly by increasing the concentration of highly oxidized Ni active sites on the catalyst surface.^7^ On the other hand, intrinsic magnetic order has also been shown to influence catalyst performance in OER, and catalytic activity has been proposed to be maximized via catalysts with dominant metallic ferromagnetic behavior.^8^

Cobalt ferrite (CFO), CoFe_2_O_4_, is a ferrimagnetic material that can exhibit spontaneous magnetization and, under appropriate conditions, such as at room temperature, can behave as a permanent magnet. Due to its good magnetic properties, i.e., saturation magnetization (*M*_s_ ≈ 80 emu/g)^9^ and first-order magnetocrystalline anisotropy constant (*K*_1_ ≈ 2 × 10^6^ ergs/cm^3^),^10^ wide availability, and relatively low cost,^11^ this material is a great candidate for large-scale applications. In addition, the magnetic properties of CoFe_2_O_4_ can be tailored toward specific applications by tuning its size and shape through the choice of synthetic strategy, such as sol–gel,^12,13^ hydrothermal methods,^14–16^ coprecipitation,^17^ and solvothermal decomposition.^18^

Herein, we employ an electrospinning template strategy to optimize the magnetic properties of CoFe_2_O_4_. The choice of the calcination temperature of the nanofibers was shown to influence both the morphology and magnetic properties, with 1173 K yielding spherical, uniformly sized particles in a nanowire-like arrangement and the highest saturation magnetization and coercivity at 2 K. The obtained CoFe_2_O_4_ material was characterized by both experimental and computational methods to shed light on its formation and the origin of the properties. Finally, magnetoelectrochemical testing for alkaline OER demonstrated the material to be a highly active catalyst, the activity of which was further significantly boosted by the application of an external magnetic field. The results highlight how efficient PGM-free OER catalysts can be obtained by capitalizing on both intrinsic magnetic order and external magnetic field.

## Results

Our approach to tailoring the magnetic properties through the microstructure of CoFe_2_O_4_ involved electrospinning of a fibrous template followed by calcination to gain access to crystalline CoFe_2_O_4_ nanomaterials. The electrospinning template strategy involved the use of an electrospinning precursor solution,^19^ the mixture consisting of three parts: Fe­(III) and Co­(II) acetyl­acetonate salts as the metal precursors in a molar ratio of 2:1, poly­acrylo­nitrile (PAN) as the polymer of choice to meet the viscosity requirements of the spinning solution, and *N*,*N*-dimethyl­formamide (DMF) as the solvent to both dissolve the metal precursors and improve the charge-carrying capacity of the solution. The optimal total metal precursor concentration of the solution was established to be 0.19 mmol of metals per mL of DMF, resulting in uniform spun nanofibers (for details, see the Experimental Section).

For the preparation of the CoFe_2_O_4_ nanowires, the resultant electrospun nanofibers were first stabilized at 573 K for 2 h under air, followed by calcination during 2 h in air at temperatures of 873, 973, 1073, 1173, and 1273 K, giving access to CFO–873 K, CFO–973 K, CFO–1073 K, CFO–1173 K, and CFO–1273 K, respectively. Fourier-transform infrared spectroscopy [FTIR; Figure S1 in the Supporting Information (SI)] showed a clear reduction in the intensity of the peaks corresponding to the nitrile functionalities at 2240 cm^–1^ and the C–H stretching vibration at 1450 cm^–1^ upon stabilization of the PAN nanofibers, followed by their expected complete disappearance after subsequent calcination.

Powder X-ray diffraction (PXRD) analysis indicated the obtained materials to be phase-pure CoFe_2_O_4_ featuring a face-centered cubic spinel structure (Figure 1a). The full width at half-maximum (fwhm) values of the diffraction peaks decreased with increasing calcination temperature (Table S1), indicating enhanced crystallization, resulting in the formation of larger and more uniform CoFe_2_O_4_ particles. The crystallite size of the synthesized CoFe_2_O_4_ was determined using the Scherrer equation:^20^

1 $$D_{\mathrm{PXRD}}=0.9\frac{\lambda }{\beta }\cos\theta$$

where *D*_PXRD_ is the crystallite size (nm), β is the full width of the diffraction line at half of the maximum intensity (measured in radians), λ is the X-ray wavelength of the used Cu *K*α = 0.15406 nm, and θ is the Bragg angle. The crystallite size estimated using the Scherrer equation expectedly increased with increasing calcination temperature (Table S2).

**Figure 1:**
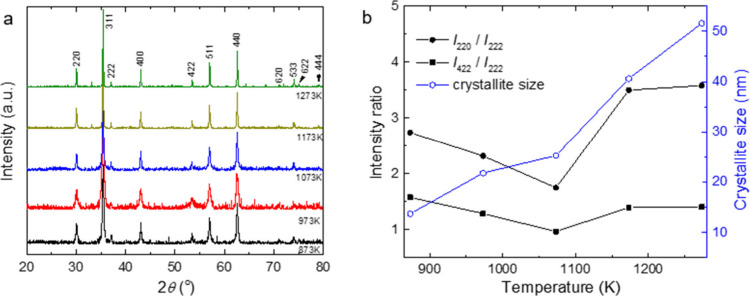
(a) Collected PXRD patterns of the CoFe_2_O_4_ nanowires obtained at different calcination temperatures. (b) PXRD intensity ratios *I*_220_/*I*_222_ and *I*_422_/*I*_222_ and crystallite size as a function of temperature.

The distribution of cations in the tetrahedral *A* and octahedral *B* sites of the spinel *AB*_2_O_4_ structure can alter the magnetic properties of the resultant CoFe_2_O_4_ nanowires. To give indications of possible differences between the samples, the intensity ratios *I*_220_/*I*_222_ and *I*_422_/*I*_222_ of the respective PXRD reflections (Table S2) were analyzed, as such analysis could hint at dissimilarities in the distribution of cations.^21–23^ While some effect of the calcination temperature was found on both intensity ratios, larger changes were found in *I*_220_/*I*_222_ (Figure 1b), ranging from 2.7 for CFO–873 K to 3.6 for CFO–1273 K. The values for CFO–1173 K, 3.5 and 1.4 for *I*_220_/*I*_222_ and *I*_422_/*I*_222_, respectively, are very close to those theoretically predicted for a system with Co^2+^ occupying only the octahedral *B* sites, which could indicate the material to form an inverse spinel crystal structure.^21^ However, for quantitative information about the cation distribution, further analysis would be required.

Scanning electron microscopy (SEM) images of the synthesized CoFe_2_O_4_ materials showed that the morphology of the as-spun nanofiber was transformed into differently shaped nanowires upon calcination at different temperatures (Figure 2a). With an increasing calcination temperature, the grain size was found to grow, and the smooth surface of the nanofibers transformed into increasing roughness. Interestingly, CFO–1173 K and CFO–1273 K featured nanoparticles merged into a chain-like morphology. The average diameter of the resultant nanowires decreased from 760 nm found for the as-spun nanofibers to approximately 184 nm for CFO–873 K, 149 nm for CFO–973 K, 140 nm for CFO–1073 K, 124 nm for CFO–1173 K, and 117 nm for CFO–1273 K (Figure 2b).

**Figure 2:**
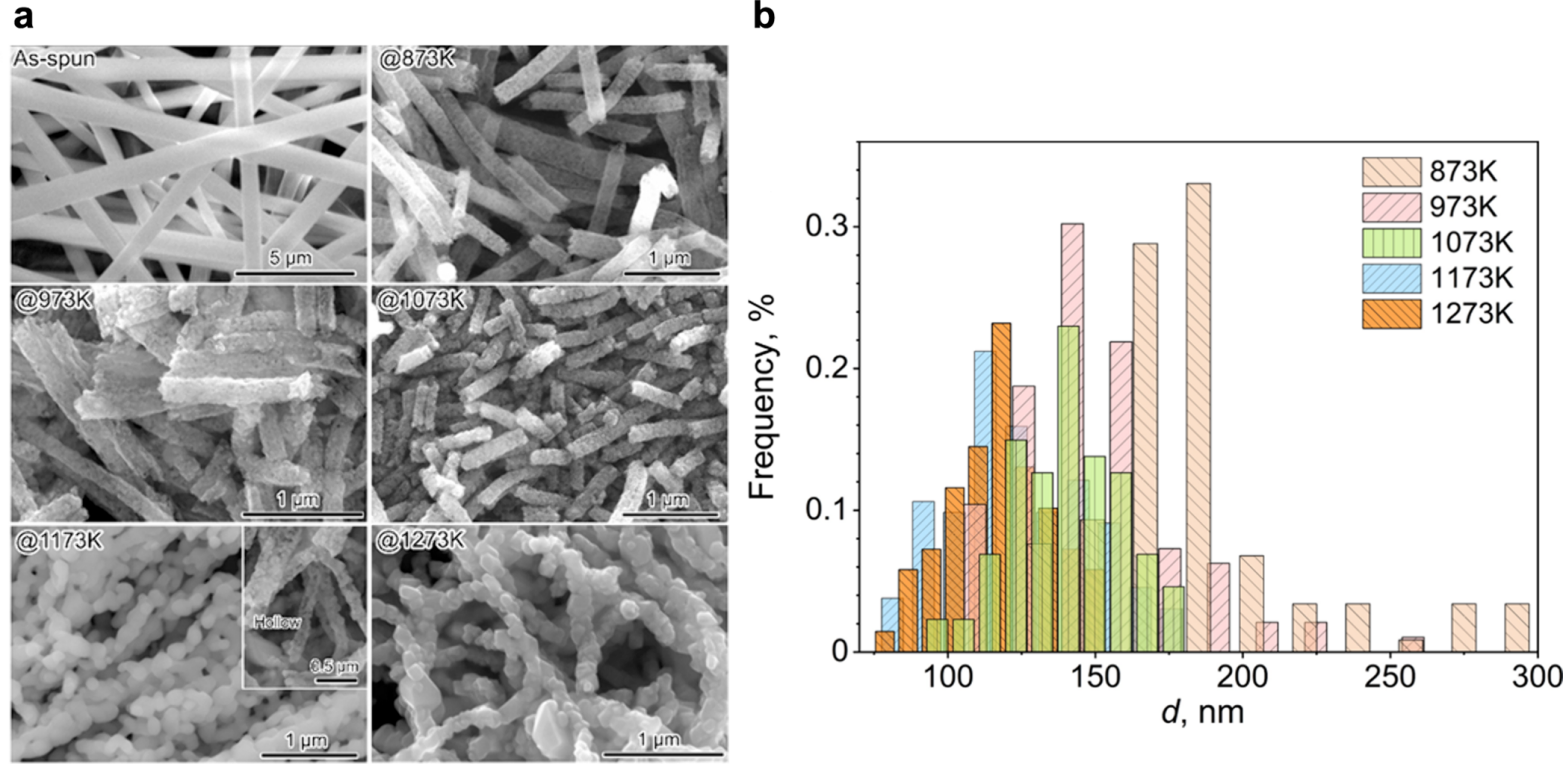
(a) Low-magnification SEM images of the as-spun Co- and Fe-containing PAN fibers, CFO–873 K, CFO–973 K, CFO–1073 K, CFO–1173 K, and CFO–1273 K. (b) Histogram of the diameters of the respective CFO nanowires after calcination.

To gain insight into the effect of the calcination temperature on the magnetic properties, the magnetization versus magnetic field *M*(*H*) dependence of the CoFe_2_O_4_ materials was measured at 2 and 300 K (Figures 3a–e). For CFO–873 K, coercivity *H*_c_ = 635 Oe was observed at 300 K. Upon increasing calcination temperature, *H*_c_ increased significantly, with CFO–1273 K showing the highest *H*_c_ = 2500 Oe (Table S3). At 2 K, substantially higher *H*_c_ values were measured, ranging from 15.9 to 17.1 kOe, with CFO–1173 K as the best-performing magnetic material. Saturation magnetization values *M*_s_ = 70–89 emu/g (2 K) and *M*_s_ = 71–93 emu/g (300 K) were estimated, with the highest value obtained for CFO–1173 K. Similarly, CFO–1173 K also showed the highest remanent magnetization *M*_r_ = 76.6 emu/g at 2 K.

**Figure 3:**
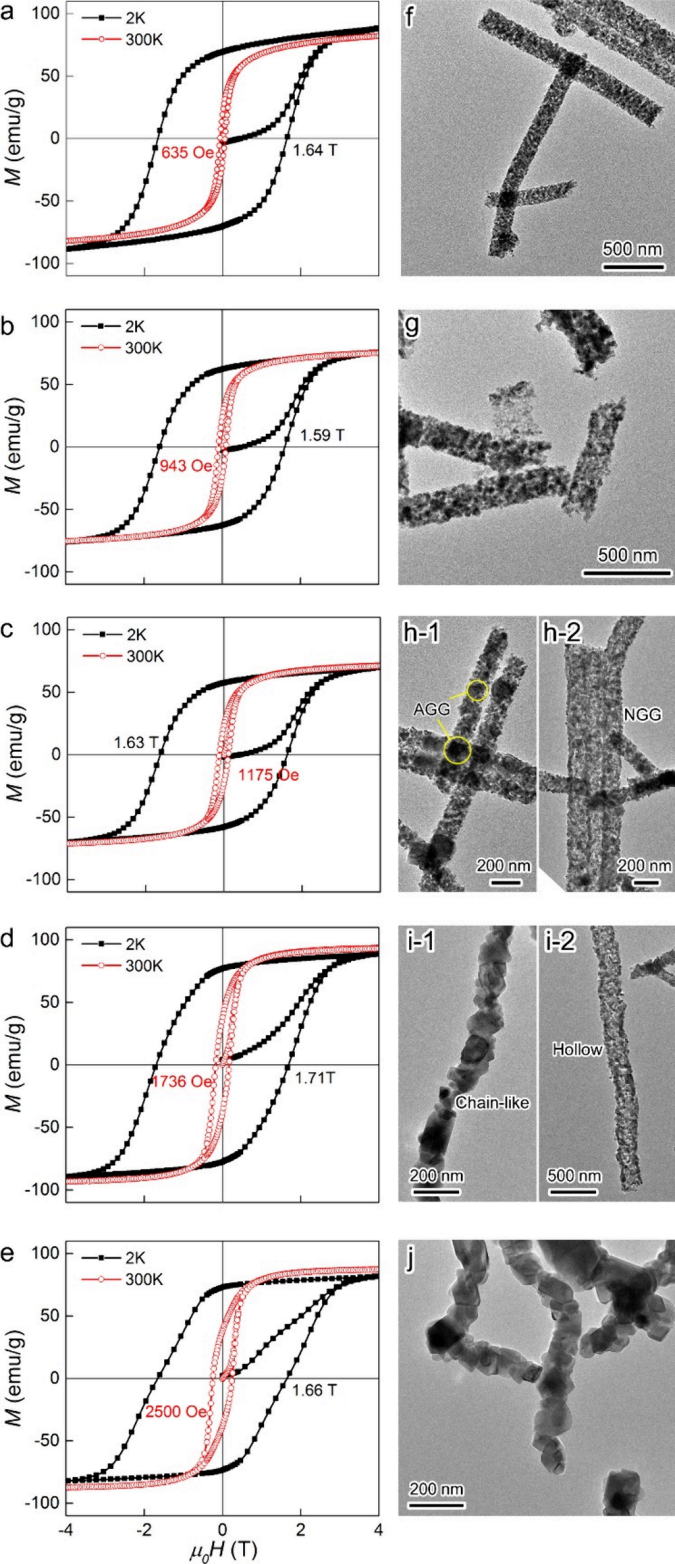
*M*(*H*) dependence data measured at 300 K and 2 K and representative low-magnification TEM images of CFO–873 K (a,f), CFO–973 K (b,g), CFO–1073 K (c,h), CFO–1173 K (d,i), and CFO–1273 K (e,j).

Transmission electron microscopy (TEM) images evidenced that CFO–873 K was composed of polycrystals with an average crystal size of 6.2 ± 2 nm (Figures 3f–j). While the increase in the calcination temperature to 973 K resulted in slight growth of the particles, for CFO–1073 K, two morphologies were observed: solid chains with partial abnormal grain growth (AGG; Figure 3h-1) as well as hollow tubes from normal grain growth (NGG; Figure 3h-2). Similar results were observed for CFO–1173 K (Figures 3i-1 and i-2), whereas the highest calcination temperature of 1273 K yielded nanowires consisting of intergrown CoFe_2_O_4_ nanoparticles (Figure 3j).

With CFO–1173 K identified as the material with the topmost magnetic properties of the prepared nanowires, i.e., the highest saturation magnetization, the largest coercivity, and the highest remanent magnetization at 2 K, a temperature indicative of intrinsic magnetic properties of a material, this sample was chosen for detailed structural characterization and subsequent magneto­electro­catalytic studies. First, intrigued by the dual nanowire structure observed in the TEM images, we studied the sample in more detail by electron microscopy to gain further insight into its fine microstructure. Microstructure analysis revealed that the nanowires consisted of CoFe_2_O_4_ crystals intergrown along the nanowire axis (Figure 4a), with the diameter of the individual particles ranging from 30 to 60 nm. Energy-dispersive X-ray spectroscopy maps recorded in scanning TEM mode (STEM-EDX) evidenced the uniform distribution of Co, Fe, and O throughout the nanowire, in agreement with the CoFe_2_O_4_ chemical composition of the material (Figure 4a). The electron hologram image showed the in-plane signals of the magnetic flux to exhibit locally centrosymmetric distribution in the individual CoFe_2_O_4_ nanoparticles (Figure 4b), confirming that CFO–1173 K nanowires are a multidomain magnetic material.

**Figure 4:**
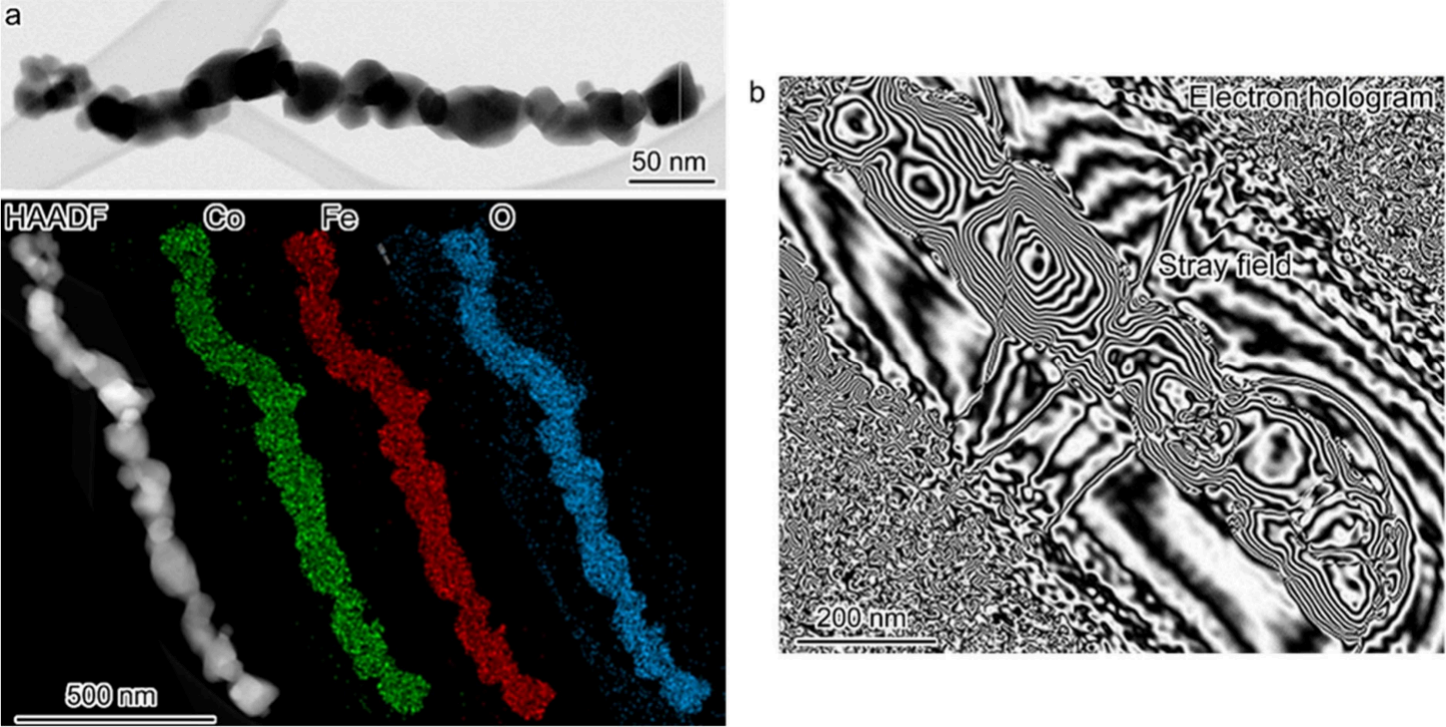
(a) Representative low-magnification TEM and HAADF-STEM images of a typical CFO–1173 K nanowire, together with the corresponding STEM-EDX maps of Co (green), Fe (red), and O (blue). (b) Typical electron holography of a magnetic CFO–1173 K nanowire.

To gain insight into the complex intergranular microstructure of CFO–1173 K, high-magnification high-angle annular dark-field scanning TEM (HAADF-STEM) was employed (Figure 5). In the selected grain boundary (Figure 5a), the areas around the transition region feature similar crystallographic orientations, and the Fourier transform (FT) patterns confirmed the presence of two grains with [011] zone axes (Figure 5b). At the interface, at least three regions (R1, R2, and R3) with different grain boundary features between the neighboring CoFe_2_O_4_ crystallites were identified. The zoomed-in HAADF-STEM images evidenced these regions to exhibit overlapped, twinning, and incoherent crystallographic relationships for R1, R2, and R3 regions, respectively (Figure 5c).

**Figure 5:**
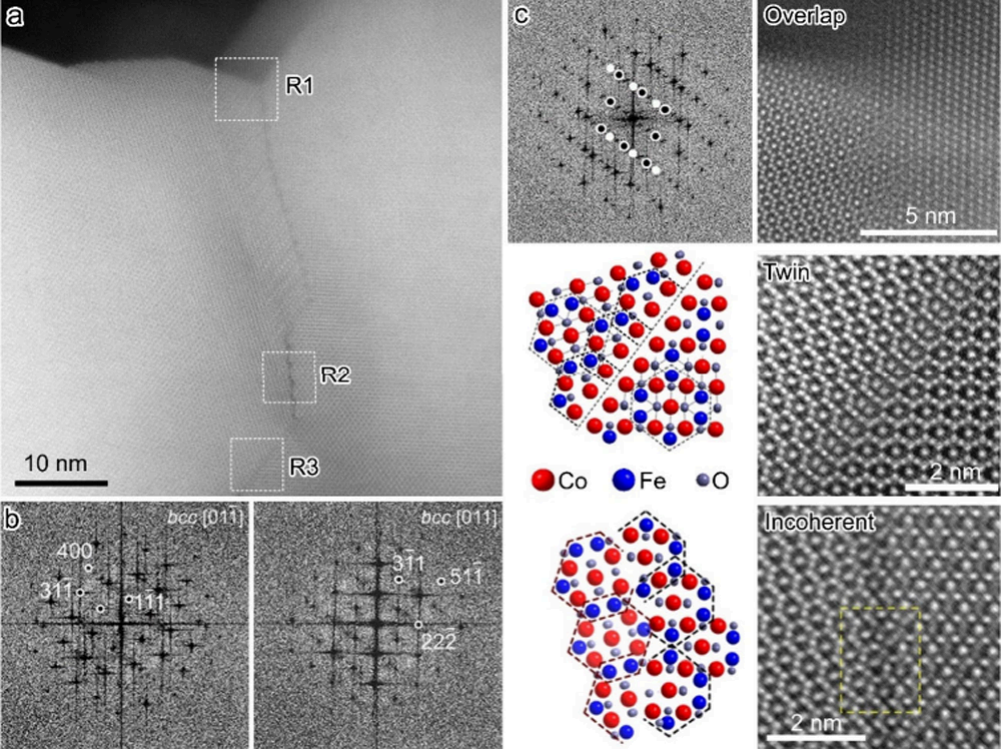
(a) Representative high-resolution HAADF-STEM image of a selected grain boundary between neighboring CoFe_2_O_4_ crystallites of the CFO–1173 K nanowire with (b) the corresponding FT patterns. (c) Zoomed-in high-resolution HAADF-STEM images of overlapped, twinning, and incoherent interfaces corresponding to regions R1–R3, respectively, in (a). FT pattern and crystal structure models are shown to describe the twin and incoherent regions.

For further structural insight into the CFO–1173 K nanowires, ^57^Fe Mössbauer spectroscopy at 6 and 293 K was employed to investigate the cation distribution in the material. The extracted Mössbauer parameters for both measurements are summarized in Table S4. Although the 6 K spectrum seemed to consist of two magnetically split sextets, careful analysis showed that the spectrum was best fitted with three sextets (Figure 6). The first two components, Q_1_ and Q_2_, with respective intensities of 36% and 15%, have the centroid shift δ values of 0.462 and 0.545 mm/s and magnetic hyperfine field *B*_hf_ values of 55.1 and 53.7 T, respectively, which are higher than the values for the third component, Q_3_. Hence, the Q_1_ and Q_2_ components are signals from the Fe atoms from the octahedral sites. The third component, Q_3_, with an intensity of 49%, has a δ value of 0.349 mm/s and a *B*_hf_ value of 51.3 T. In agreement with the PXRD data above, these results indicate that the cation distribution in the resultant spinel *AB*_2_O_4_ structure, within the experimental error, is [Fe]^A^{CoFe}^B^O_4_, where *A* is for the tetrahedral sites and *B* is for the octahedral sites; i.e., Fe atoms are evenly distributed in the tetrahedral and the octahedral sites, as expected for an inverse spinel structure,^24^ and hence Co atoms occupy only the octahedral sites. As there is no signal for Fe^2+^, all of the Co atoms will be in the Co^2+^ oxidation state to preserve overall neutrality. The room-temperature Mössbauer spectrum for CFO–1173 K, although with a changed pattern, was also best fitted with three sextets. While the *B*_hf_ values for Q_1_ (*B* site) and Q_3_ (*A* site) decreased by 2.8 and 2.1 T, respectively, the *B*_hf_ value for Q_2_ (*B* site) decreased by 3.9 T.^25–30^

**Figure 6:**
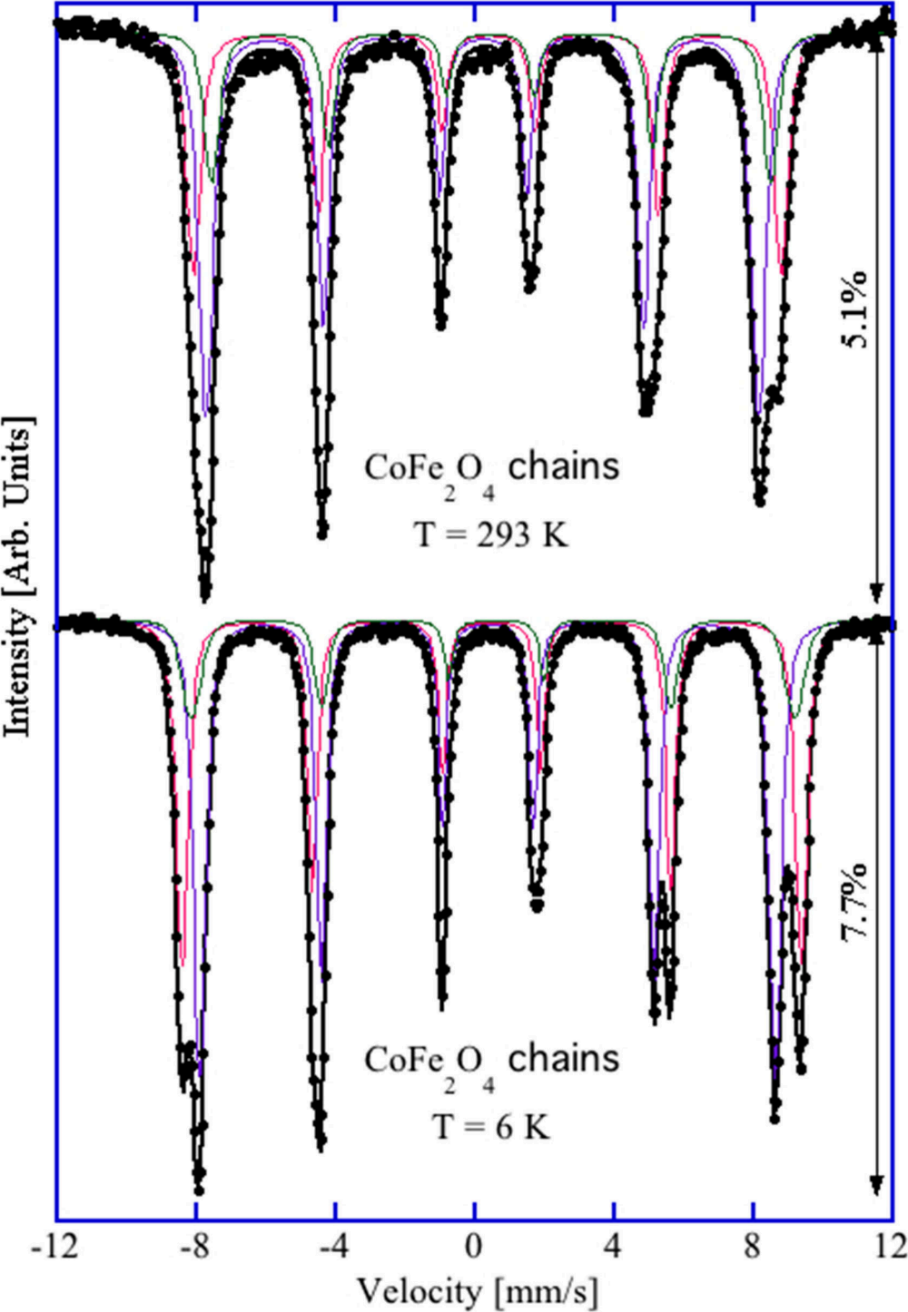
Room-temperature (top) and low-temperature (bottom) ^57^Fe Mössbauer spectra for CFO–1173 K nanowires. Experimental data are shown as black circles, while calculated spectra are shown as black lines. Q_1_, Q_2_, and Q_3_ components are shown in red, green, and violet, respectively.

For further characterization of the intrinsic magnetic properties of CoFe_2_O_4_, DFT+U+J (Dudarev) calculations were carried out (Section S2 in the SI; Figures S2–S5). A ferrimagnetic inverse spinel CoFe_2_O_4_ bulk structure^31^ was used as the model system to test the performance of two GGA functionals, PBE^32^ and optB86b,^33^ with and without semiempirical dispersion corrections (DFT-D3)^34^ for the former and with a van der Waals nonlocal term^35^ for the latter. The outcome of the screening indicated that the optB86b functional without the inclusion of the van der Waals nonlocal term and a *U*_eff_ (*U*_eff_ = *U* – *J*) of 3.6 eV for Fe atoms (*U* = 4.0 eV and *J* = 0.4 eV) and 2.5 eV for Co (*U* = 3.0 eV and *J* = 0.5 eV) atoms was the combination giving the best agreement with the experimental data (see Tables S6 and S7 in the SI). Thus, the optB86b+U+J approach was selected to computationally characterize the CFO nanowires.

A surface slab model of ferrimagnetic CFO–1173 K, the material with the highest *M*_s_, was built combining the cation distribution [Fe]­{CoFe}­O_4_ obtained from ^57^Fe Mössbauer spectroscopy (Figure 6) and HAADF-STEM imaging (Figure 5) data (see SI for further details). The calculated intrinsic magnetic properties of bulk CFO and (011) CFO–1173 K structures are summarized in Figure 7. Indeed, the estimated computational magnetic moment per formula unit (f.u.) of ferrimagnetic (011) CFO–1173 K slab model is 3.34 μ_B_ (spin-only component) at 0 K, which is in decent agreement with the experimental value of 3.74 μ_B_ obtained from saturation magnetization measurement with *M*_s_ = 88.9 emu/g at 2 K (Tables S3 and S9). The improved magnetic properties of (011) CFO–1173 K are also confirmed by the increment of ∼10% in the magnetic moment per f.u. with respect to the CFO bulk value of 3.04 μ_B_, similarly to the experimental saturation magnetizations *M*_s_ = 88.9 emu/g of CFO–1173 K and *M*_s_ = 80.8 emu/g^36^ of bulk CFO.

**Figure 7:**
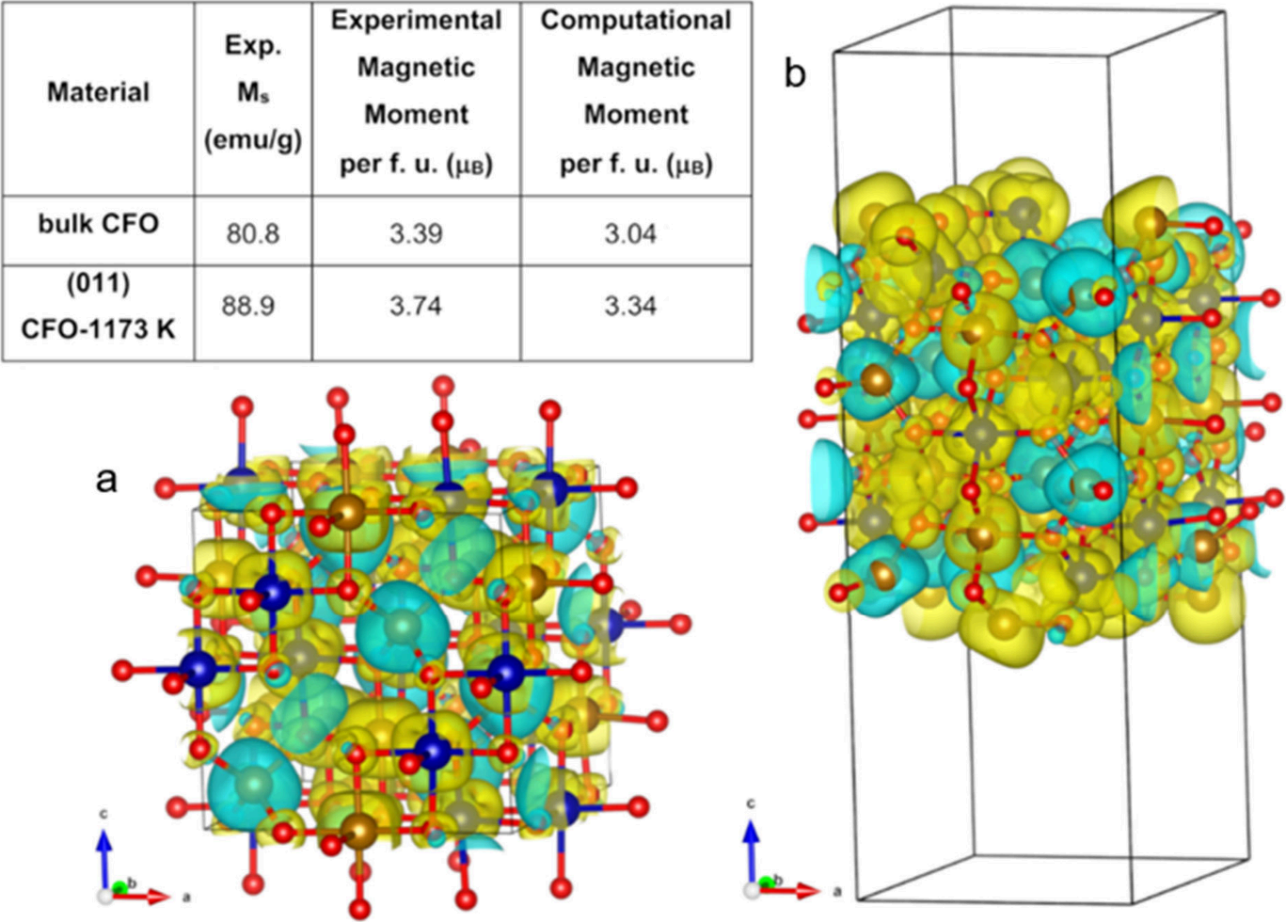
Comparison between the experimental and calculated magnetic moments per formula unit (spin-only component) of bulk CFO and synthesized (011) CFO–1173 K. Spin density plots of ferrimagnetic inverse spinel bulk CFO (a) and the (011) CFO–1173 K slab model (b). Spin-up and spin-down densities are colored yellow and cyan, respectively. The isosurfaces are shown at 0.004 *a*_0_^–3^ (*a*_0_ is the Bohr radius).

With the structural information on CFO–1173 K in hand and considering its topmost magnetic properties, we next studied its electrocatalytic activity in alkaline OER. The recent increasing interest in magneto­electro­chemistry, and particularly harnessing magnetic effects for the enhancement of the OER performance of catalysts, underlines the importance of reliability in magneto­electro­chemical measurements. The stability of the catalytic performance under the OER conditions must be ensured; hence, merely reporting electrochemical activity results is not sufficient. The use of an external magnetic field may result in an increase in the temperature, and therefore its monitoring is crucial to exclude that changes in catalyst performance occur due to changes in temperature. Furthermore, possible changes in the electrochemically active surface area upon subjecting the catalysts to an external magnetic field should be excluded as the reason for enhanced OER activity.

The OER activity of CFO–1173 K was studied by cyclic voltammetry (CV) and linear sweep voltammetry (LSV) in a three-electrode cell (Figure S6; Section S3 in SI). The OER activity was also examined under an external magnetic field (Figure 8) using a custom dipole electromagnet capable of fine-tuning the field strength from *H* = 0 to *H* = 500 mT (Section S4 in the SI). Importantly, this work provides a reliable approach for conducting magneto­electro­chemical measurements, negating the temperature and surface area effects.

**Figure 8:**
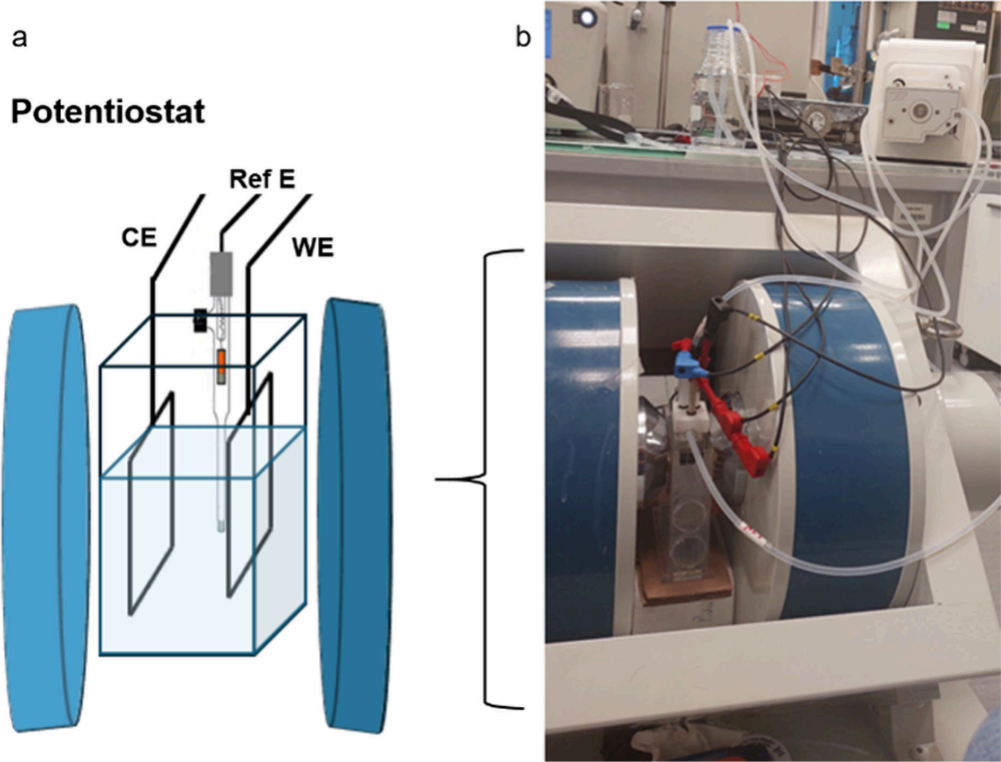
Schematic (a) and laboratory (b) electromagnet and electrochemical cell setup equipped with a water circulation thermostation (CE = counter electrode; ref E = reference electrode; WE = working electrode).

CFO–1173 K powder was formulated as an ink in ethanol with a conductive Nafion ionomer as binder, and the ink was deposited onto Ni felt (Bekaert). Experimentally, the nanowires were found to demonstrate the best OER performance at 1 mg/cm^2^ mass loading (data not shown). The measurements under an external magnetic field were carried out after those in the absence of the field. After 2 min at *H* = 500 mT, LSV curves were determined up to the number of cycles where no further increase in the current density was found as compared to the results in the absence of the external magnetic field (*H* = 0 mT). Notably, if no increase or decrease was found in the presence of the external magnetic field, the direction of the field was changed by rotating the working electrode by 180°. Further details on the measurements in the absence and presence of the external magnetic field can be found in the SI.

CFO–1173 K showed high activity as an OER catalyst (Figures 9a,b; Table S11), reaching current densities *j* of 10, 20, 50, and 100 mA/cm^2^ at overpotentials η of approximately 318, 341, 386, and 448 mV, respectively. Remarkably, the application of an external magnetic field was found to result in a reduction in the required overpotential at all current densities, with a greater reduction observed with increasing current density from 26 mV at 10 mA/cm^2^ (decrease of 8%) to 95 mV at 100 mA/cm^2^ (decrease of 21%). The Tafel slope was found to decrease slightly from 50 to 43 mV/dec upon introduction of the magnetic field (Figure 9c), suggesting that no drastic changes occur in the mechanism or rate-determining step during the OER. The activity of CFO–1173 K was also compared to that of the control Ni felt current collector, as well as those of commercial benchmark PGM-based IrO_2_ (TKK, TEC77100) and PGM-free NiFe_2_O_4_ (20 nm, US Research Nanomaterials) catalysts (Figure S7), which showed overpotentials of 430, 275, and 343 mV, respectively, at a current density of 100 mA/cm^2^ (Table S12). As expected, the activity of IrO_2_ did not improve upon application of an external magnetic field, whereas NiFe_2_O_4_ and Ni felt showed a reduction in the required overpotential of 16 and 43 mV at 100 mA/cm^2^, respectively.

**Figure 9:**
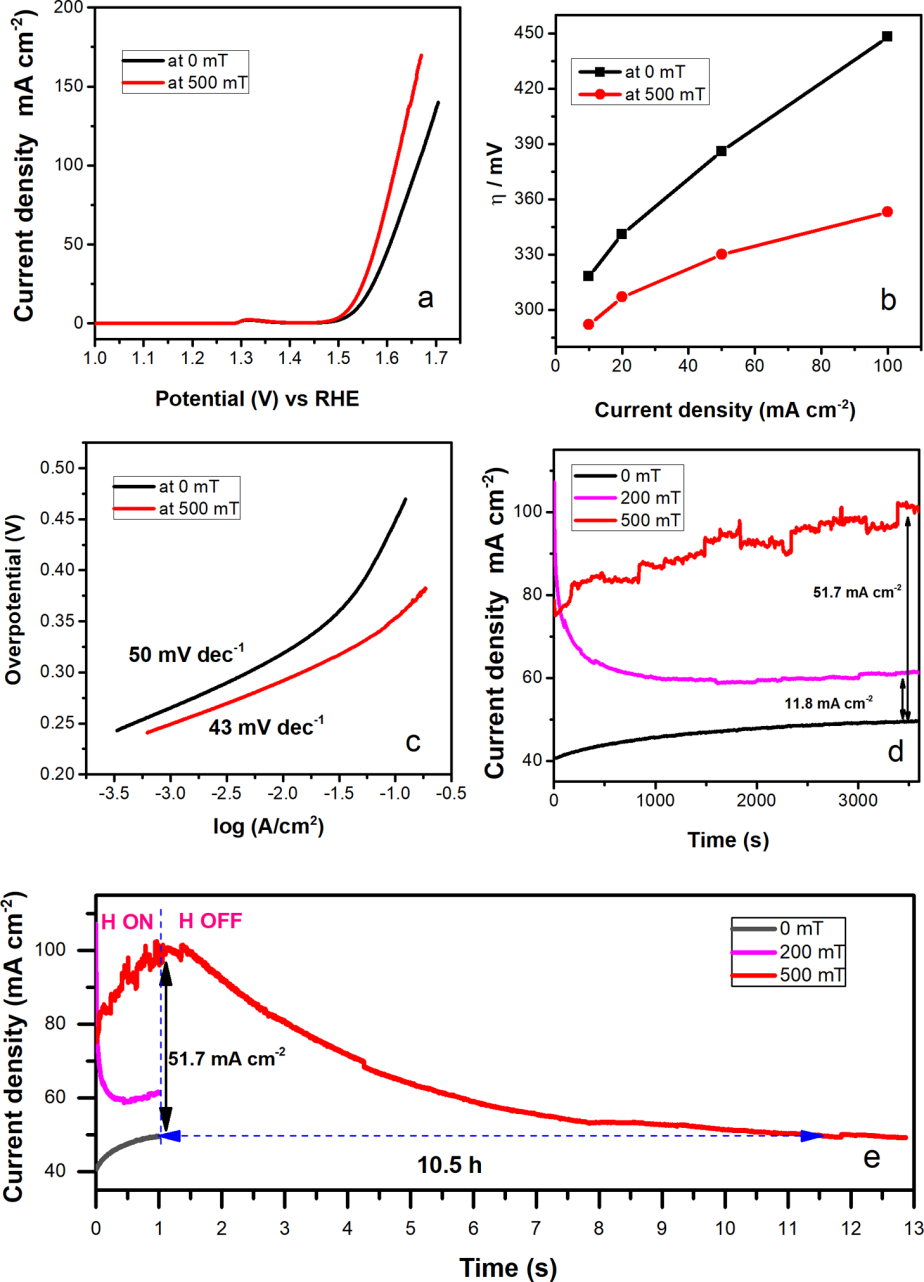
Magnetoelectrochemical data of CFO–1173 K with (*H* = 500 mT, red; *H* = 200 mT, magenta) and without (*H* = 0, black) an external magnetic field: (a) LSV at the scan rate of 5 mV/s, (b) overpotentials at current densities of 10, 20, 50, and 100 mA/cm^2^, and (c) Tafel plots extracted from the LSV data. (d) Using a demagnetized sample, chronoamperometry curves during 1 h at 1.6 V_RHE_. (e) Chronoamperometry curves during 13 h at 1.6 V_RHE_ (*H* refers to an external magnetic field). All were recorded in 1 M KOH at room temperature. All polarization curves are 85% *iR*-compensated.

To exclude that the increase in the catalytic performance under magnetic field stems from changes in the electrochemically active surface area, cyclic voltammograms were recorded with different scan rates (5 to 70 mV/s) in the non-faradaic region with and without applied magnetic field (Figures S8a,b). The geometric double-layer capacitance, *C*_dl_, obtained from plotting the average current density at mid-potential against the scan rate (Figure S8c), increases slightly from 1150 μF/cm^2^ without magnetic field to 1350 μF/cm^2^ in the presence of the external magnetic field of 500 mT. Normalizing OER anodic polarization curves in Figure 9a to the measured geometric double-layer capacitance (Figure S9) indicated that the enhancement caused by the external magnetic field cannot be attributed to an increased electrochemically active surface area.

To remove the effect of any unknown remanent magnetization of the sample, CFO–1173 K was demagnetized by applying an alternating magnetic field until the magnetization reached zero (Figure S10). We then carried out chronoamperometric measurements at a constant potential of 1.6 V_RHE_ during 1 h at *H* = 0, 200, and 500 mT. The results also illustrated the increase in the catalytic performance under magnetic field, with enhancements of current density of 11.8 mA/cm^2^ at 200 mT and 51.7 mA/cm^2^ at 500 mT, the latter corresponding to an impressive increase of 100% (Figure 9d). During 13 h testing (Figure 9e), where a magnetic field (*H*_ON_) was applied during the initial 1 h, an increase in the catalytic activity was retained for up to 10.5 h after removing the magnetic field, reflecting the influence of the good remanent magnetization properties of CFO–1173 K.

Finally, for comparison, we also measured the catalytic activity of CFO–1273 K, a sample with slightly lower coercivity and saturation magnetization at 2 K as compared to CFO–1173 K. While in the absence of an external magnetic field the performance of the materials was comparable (Figure S11), upon application of an external magnetic field of 500 mT CFO–1273 K showed a magneto­current of 33.4 mA cm^–2^ (at 1.67 V_RHE_), which corresponds to an enhancement of only 68%. This highlights the importance of the intrinsic magnetic properties for the enhancement of the magneto­electro­chemical activity.

## Discussion

Electrospinning can be employed to gain access to magnetic nanofibers via three common strategies: supporting magnetic particles on electrospinning polymer materials, postdeposition on electrospun polymer surfaces, and an electrospinning template strategy^37^ followed by calcination. We opted for the latter, as it prevents dilution of the magnetic properties by the polymetric matrix and can yield nanomaterials with good magnetic properties.^38^ The heating rate was previously demonstrated to have an effect on both the crystallinity and magnetic properties of CoFe_2_O_4_ nanofibers,^39^ with slower rates resulting in enhanced properties at higher calcination temperatures. The effect of calcination temperature on the cation distribution has been studied using CoFe_2_O_4_ nanofibers electrospun from polyvinyl­pyrrolidone.^23^ With increasing temperature, Co^2+^ ions were found to shift to the tetrahedral *A* sites and some Fe^3+^ cations to the octahedral *B* sites of the spinel *AB*_2_O_4_ structure, resulting in enhanced saturation magnetization of up to 83 emu/g. In a study using sol–gel electrospinning, the best-performing CoFe_2_O_4_ nanofibers featured saturation magnetization of 76 emu/g and coercivity of 723 Oe at room temperature, with the good magnetic properties attributed to the microstructure of the material featuring grains arranged in a linear chain configuration.^40^ In our study, electrospinning of the PAN solution in DMF containing the metal precursors gave access to smooth nanofibers, and optimized calcination conditions resulted in CoFe_2_O_4_ nanowires with an average diameter of around 120 nm for CFO–1173 K, featuring morphologies of solid chains as well as hollow tubes.

Based on the insight into the microstructure of the CoFe_2_O_4_ nanowires obtained by TEM, we propose the microstructure of the CoFe_2_O_4_ nanowires to evolve in a temperature-dependent manner as depicted in Figure 10. All of our proposed transformations start with as-spun well-defined and smooth Co- and Fe-containing PAN nanofibers. A key aspect of CoFe_2_O_4_ preparation is the calcination-assisted elimination of the PAN template. This occurs in two phases: a low-temperature stabilization thermal treatment conducted at 573 K in air, essential to avoid disintegration of the PAN nanofibers, and subsequent burning of PAN at 873–1273 K. A slow heating rate of 1 °C/min to 973 K resulted in homogeneous nucleation of CoFe_2_O_4_ crystals and uniform grain growth, resulting in solid rods consisting of polycrystalline spinel particles. At higher calcination temperatures in the range between 1000 and 1173 K, diffusion through the Kirkendall effect^41–43^ in some of the nanofiber precursors led to the formation of hollow-fiber nanotubes. Meanwhile, in part of the CoFe_2_O_4_ nanowires, local abnormal grain growth resulted in nanowires with both coarse- and fine-grained solids. At the calcination temperature of 1273 K, the heterogeneous grain growth mechanism seems to dominate microstructure formation, resulting in nanowires consisting solely of large, intergrown nanoparticles.

**Figure 10:**
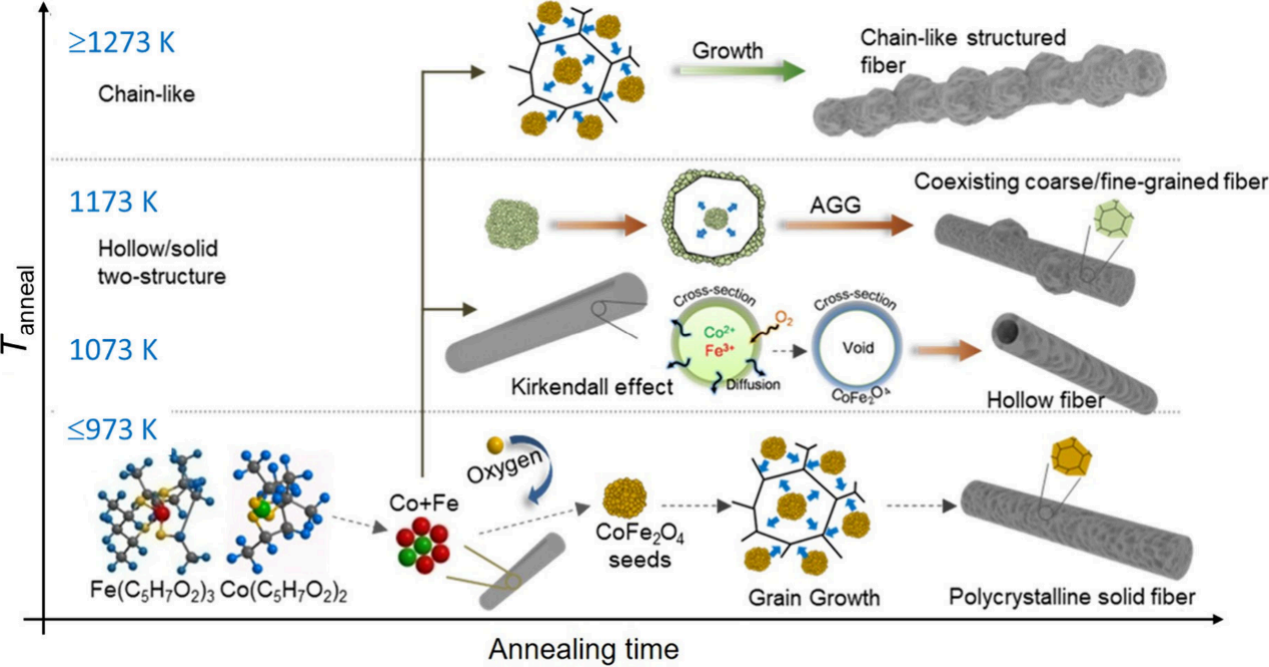
Schematic illustrations of the temperature-dependent evolution of the microstructure of the synthesized CoFe_2_O_4_ nanowires. At calcination temperatures below 1000 K, Co^2+^/Fe^3+^ cations from metal–organic precursors react with oxygen to form solid polycrystalline CoFe_2_O_4_ nanofibers with rough surfaces. At calcination temperatures in the range between 1000 and 1200 K, the nanowires feature a coexisting hollow nanotube appearance and polycrystal solid nanorod microstructures. In the hollow fibers, calcination results in the formation of hollow CoFe_2_O_4_ nanotubes by interfacial solid-state diffusion induced by the Kirkendall effect. In the solid fibers, abnormal grain growth (AGG) of some fine grains leads to the formation of large, coarse grains. At calcination temperatures over 1200 K, chain-like coarse grains of CoFe_2_O_4_ are arranged as nanowires.

To gain insight into the effect of the calcination temperature on the magnetic properties of the cobalt ferrite nanowires, we plotted (Figure S12) the intensity ratios *I*_220_/*I*_222_ and *I*_422_/*I*_222_ from the PXRD data (cyan and yellow histograms, respectively), the crystallite size in nm (red line), saturation magnetization (blue line) and remanent magnetization (pink line) in emu/g, and tendency of coercivity at 300 K in Oe (red arrow). As the PXRD and Mössbauer data indicated that CFO–1173 K is an inverse spinel, its inversion parameter δ is equal to 1. This information was used as a reference to estimate the inversion parameters of the nanowires prepared at different calcination temperatures. The figure illustrates that the crystallite size and coercivity of the nanowires increase with increasing calcination temperature, independent of the inversion parameter. In contrast, the variations of the saturation and remanent magnetization do not appear to be directly related to the changing calcination temperature. The highest saturation and remanent magnetization are featured by inverse spinel CFO–1173 K and CFO–1273 K, indicating a relationship among these magnetic properties, the cation distribution, and the crystallite size. The inversion parameter does not appear to linearly correlate with the calcination temperature; however, high calcination temperatures seem to favor an inverse cation distribution with *δ* = 1.

The excellent magnetic properties of CFO–1173 K are evidenced by the high saturation magnetization of 88.9 emu/g at 2 K (92.6 emu/g at 300 K), surpassing other reported electrospun CoFe_2_O_4_ nanofibers (56.5 emu/g at 300 K;^44^ 69.46 emu/g^45^), CoFe_2_O_4_ nanotubes (80 emu/g at 300 K^46^), and bulk CoFe_2_O_4_ (80.8 emu/g).^36^ Furthermore, the high coercivity values (17.1 kOe at 2 K; 1736 Oe at 300 K), which we attribute to the large grain size in the nanowires, were significantly higher than those reported for electrospun CoFe_2_O_4_ nanofibers (960 Oe at 300 K^44^), nanotubes (300 Oe at 360 K^46^), or nanoribbons (900 Oe^47^), highlighting the ferrimagnetic character of the material.

OER is a complex multielectron process, with a scaling relationship for the binding of *OOH, *O, and *OH intermediates hindering the optimization of catalysts.^48^ Recently, magneto­electro­chemical effects are raising increasing interest as means to overcome these relationships, not only through indirect effects such as local heating or Lorentz forces^49^ but via spin alignment,^7^ where the intrinsic magnetic order of the catalyst and/or an external magnetic field can promote the formation of triplet state oxygen from singlet state reaction intermediates. However, the origins of the improvement of oxygen electrocatalysis under magnetization are still not completely understood.

To fully exploit spin-polarization effects in magnetoelectrochemical OER, reaction pathways of the OER should be considered. In lattice OER (LOER), the lattice oxygen of the catalyst oxidizes, which is accompanied by continuous dissolution–redeposition of metal cations and leads to O_2_ evolution.^50,51^ The adsorbent evolution mechanism (AEM) involves the formation of the *OOH intermediate through an attack on the *O species, whereas the related intermolecular mechanism (I2M) pathway features the M–O–O–M intermediate stemming from the coupling of M–O species from neighboring active sites.^52^ Consequently, an atomic-level understanding of the influence of the catalytic mechanism on the magnetic enhancement of the OER is of importance. To this end, Xu and co-workers^52^ reported three model Ni catalysts supported on multidomain Fe_3_O_4_ to show varying OER improvement by magnetization, for which the highest enhancement was found for the Ni shell catalysts, while Ni clusters and isolated Ni sites showed much lower magnetic enhancement of OER activity. This was attributed to the fact that, upon magnetization of Fe_3_O_4_, the spins of the ligand oxygens in the Ni shell align in the direction of the domains of the support, facilitating O–O coupling for subsequent triplet O_2_ formation via the I2M pathway. In addition, lattice oxygen was proposed to participate by creating additional spin-polarized oxygen intermediates. On the other hand, the spin structure of the isolated Ni sites is less affected by Fe_3_O_4_ spin polarization in the AEM pathway, thus explaining the lower OER enhancement through magnetization of these catalysts.

Ferromagnetic ordering enhances catalytic activity,^8^ but the influence of an external magnetic field on the catalytic activity has been shown to depend on the magnetic domain structure of the catalyst material.^53^ Ferromagnetic single-domain catalysts showed spin-polarized OER performance without an external magnetic field and a negligible activity increment upon its application, while for multidomain particles an external magnetic prompt resulted in a clear activity enhancement. Similar results were recently reported for Fe_7_S_8_ nanosheets.^54^ This was attributed to the already ordered spin configurations of single-domain particles, highlighting that no external magnetic field is required in such cases.^53^ We found this to be the case for our superparamagnetic spinel ferrites, for which increasing saturation magnetization correlated with the OER activity of the catalyst.^24^

To gain insight into the mechanism of activity enhancement by an external magnetic field of multidomain materials, Xu and co-workers studied multidomain NiFe thin films of different thicknesses and observed the most improvement in OER with an applied external magnetic field for the films with the largest proportion of magnetic domain walls.^55^ This correlation was attributed to disordered spins in the domain walls, which, when subjected to an external magnetic field, orient to form a single-domain state. Thus, the degree to which the OER can be enhanced by magnetization in these materials is determined by the amount of domain walls that can be reformatted to a single magnetic domain.

In the case of non-ferromagnetic catalysts, the spin-pinning effect can be exploited to benefit from spin ordering through magnetism, as was shown with ferromagnetic oxide/oxyhydroxide catalysts.^56^ The Co_3–*x*_Fe_*x*_O_4_ catalyst with a thin reconstructed oxyhydroxide layer outperformed the as-prepared CoFe oxyhydroxides by an order of magnitude, and the activity of the former was further boosted by aligning the pinned spins of the oxyhydroxide layer with those of the ferromagnetic substrate through an external magnetic field.

The magnetic properties of our CFO–1173 K thus rendered the material highly promising in terms of its potential as an active OER catalyst. An increase in coercivity, for example, has been shown to directly enhance the spin-polarized oxygen evolution under a magnetic field with single domain particle size in the 10–40 nm range.^57^ Confirming the effect of the external magnetic field on the multidomain CFO–1173 K with high coercivity, we found a 95 mV decrease in the overpotential required at 100 mA/cm^2^, comparing favorably to literature-reported FeO_*x*_ (10 mV) and NiZnFe_4_O_*x*_ (30 mV).^6^ In the presence of an external magnetic field of *H* = 500 mT, the current was found to increase from 89.5 to 142.0 mA/cm^2^ at 1.65 V_RHE_. On the other hand, the Tafel slope at 500 mT was found to decrease by 7 mV/dec to 43 mV/dec, indicating that while reaction kinetics are slightly improved,^58^ no changes occur in the rate-determining step upon application of the magnetic field.

With CoFe_2_O_4_ as OER catalyst, it has been theoretically shown that Co and Fe sites can play a synergetic role and coexist as active sites, with Co sites favoring the adsorbate evolution mechanism and Fe sites the lattice oxygen evolution mechanism.^59^ Interestingly, spin sensitivity has been proposed for all oxygen evolution mechanisms—LOM, AEM, and I2M.^60^ In LOM and I2M, spin polarization facilitates the coupling of two M–O* species, whereas in AEM, spin polarization can facilitate the O–O coupling by an attack of OH^–^ or H_2_O on M–O*. To gain a deeper understanding of the mechanism of the OER over CFO–1173 K, detailed mechanistic studies are required, which will be the subject of a following study. In the case of our multidomain ferrimagnetic CFO–1173 K, the activity increase of 100% observed with an external magnetic field of 500 mT most likely stems from ordering of the spin configurations of the domain walls to a single-domain structure,^55^ resulting in spin-polarization-promoted OER. The small increase in *C*_dl_ further indicates that the observed enhancement in the OER activity with external magnetic field stems predominantly from spin polarization,^52^ ruling out other mechanisms like Lorentz forces and magneto­hydrodynamic effect.^49,61^ Furthermore, the chronoamperometry results under a magnetic field evidenced the benefits of the high remanent magnetization of CFO–1173 K, where the increase in the current density was retained for the studied 10.5 h after removal of the external magnetic field. This performance ranks our CFO–1173 K among the best-performing catalyst materials in terms of magnetocurrent (Table S13) and demonstrates the promise of tailoring magnetic properties of catalysts to boost efficiency in magnetoelectrochemistry. Considering that in anion exchange membrane water electrolyzers operating below 2.0 A/cm^2^ a major loss stems from OER overpotential,^62^ taking advantage of the magnetic properties of catalysts offers an attractive means to enhance the energy efficiency of green hydrogen production.

## Conclusions

An electrospinning template strategy was employed for tailoring the magnetic properties of CoFe_2_O_4_. The formation mechanism of the resultant anisotropic structures was elucidated by means of electron microscopy, revealing temperature-dependent trends. A calcination temperature of 1173 K gave CoFe_2_O_4_ nanoparticles intergrown into a nanowire-like arrangement, which exhibited high saturation magnetization of 88.9 emu/g and coercivity of 17 100 Oe at 2 K. The material was shown to be a highly active OER catalyst in alkaline electrolytes, with an overpotential of 318 mV at a current density of 10 mA/cm^2^. Interestingly, catalytic activity was boosted further by ∼100% via judicious employment of an external magnetic field. Furthermore, this activity boost was found to remain for up to 10.5 h after removal of the magnetic field. The results obtained highlight how efficient PGM-free catalysts for magnetoelectrochemistry can capitalize on both intrinsic magnetic order and an external magnetic field.

## Experimental Section

### Electrospinning

Iron­(III) acetylacetonate (Fe­(acac)_3_, 1.0 mmoL) and cobalt­(II) acetylacetonate (Co­(acac)_2_, 0.5 mmol) were dissolved in *N*,*N*-dimethylformamide (DMF, 8 mL) under vigorous stirring. Polyacrylonitrile powder (PAN, average *M*_w_ = 150,000; Sigma-Aldrich) was added to the Fe^3+^/Co^2+^/DMF solution to give a concentration of 11%. Before electrospinning, the solution was stirred at 80 °C for 6 h to obtain a transparent homogeneous solution. During electrospinning, the nanofibers, obtained at a voltage of 22 kV with a spinning distance of 18 cm and a feed rate of 0.5 mL/h, were collected on aluminum foil.

### Calcination

The as-spun Co- and Fe-containing PAN nanofibers were first stabilized at 573 K for 2 h under air and then calcined in air at various temperatures (873, 973, 1073, 1173, and 1273 K) at a heating rate of 1 °C/min for 2 h to give CFO–873 K, CFO–973 K, CFO–1073 K, CFO–1173 K, and CFO–1273 K, respectively.

### Computational Study

More details about the computational methods employed can be found in SI Section S2.

### Magnetoelectrochemistry Study

Experimental details for the conventional electrochemical and magnetoelectrochemical OER studies are available in SI Sections S3 and S4, respectively.

## Supplementary Material



## Notes

Characterization methods, computational study, and electrochemical measurements, as well as data from magnetic and Mössbauer measurements and OER performance of the materials (PDF)
