# Supplementary material for: Electrospun CoFe2O4 Nanowires Tailored for Magnetoelectrochemistry
Source: ACS Nano. 2025 Aug 6;19(32):29195–206. doi: 10.1021/acsnano.5c03628 (PMC12368993; doi:10.1021/acsnano.5c03628)
Supplement: Supplementary file 1 [file nn5c03628_si_001.pdf]

Supporting Information for

Electrospun $CoFe_2O_4$ Nanowires Tailored for Magnetoelectrochemistry

Zexuan Wang,[†] María F. Navarro Poupard,[†] Ramsundar Rani Mohan,[†] Loukya Boddapati,[†] Jijun Zhang,[†] Saeed Kamali,[≠,⁄] Chiara Biz,[Ψ] Mauro Fianchini,[Ψ,Ω] Francis Leonard Deepak,[†] Jose Gracia,[Ψ] Laura M. Salonen,[⊥,†] Yury V. Kolen'ko[†]*

[†] International Iberian Nanotechnology Laboratory, Braga 4715-330, Portugal

[≠] Department of Physics and Astronomy, Middle Tennessee State University, Murfreesboro, TN 37132, United States of America

[⁄] Department of Mechanical, Aerospace and Biomedical Engineering, University of Tennessee Space Institute, Tullahoma, TN 37388, United States of America

[Ψ] MagnetoCat SL, Calle General Polavieja 9, 3Izq, Alicante 03012, Spain

[Ω] Departamento de Química Física, Universidad de Alicante, Carretera San Vicente del Raspeig s/n, 03690 San Vicente del Raspeig, E-03080 Alicante, Spain

[⊥] CINBIO, Universidade de Vigo, Department of Organic Chemistry, 36310 Vigo, Spain

Corresponding Author: Yury V. Kolen'ko, yury.kolenko@inl.int

Section S1. Characterization

Fourier transform infrared spectroscopy (FTIR) measurements were performed on Vertex 80 v spectrometer (Bruker). Powder X-ray diffraction (PXRD) was conducted on a X'Pert PRO diffractometer (PANalytical) equipped with Cu K_α radiation ($\lambda = 1.541874$ Å) and a PIXcel detector. Data were collected using Bragg–Brentano geometry in the $2\theta = 20–80°$ range with a scan speed of 0.02°/s. The PXRD patterns were matched to International Centre for Diffraction Data (ICDD) PDF-5 database using HighScore software package (PANalytical). The morphology of the samples was studied by scanning electron microscopy (SEM) using a Quanta 650 FEG ESEM microscope (FEI). Transmission electron microscopy (TEM), high-angle annular dark-field scanning TEM (HAADF−STEM), energy-dispersive X-ray spectroscopy in STEM mode (STEM−EDX) and electron holograms were carried out on JEM-2100 (Jeol), Titan ChemiSTEM 80–200 kV (FEI), and Titan Themis 60−300 kV (FEI) electron microscopes. Magnetic measurements were carried out on powder samples by using a superconducting quantum interference device (SQUID) magnetometer (Quantum Design). ^{57}Fe Mössbauer spectroscopy measurements were performed using a MS4 spectrometer (SEE Co.) operating in the constant acceleration mode in transmission geometry. The measurements were performed at 6 K and 293 K. A 50 mCi ^{57}Co in Rh held at room temperature (RT) was used as the source. All centroid shifts, δ, are given with respect to metallic α−Fe at RT. The spectra were least square fitted by a Voight-based approach using the Recoil software[1] to extract hyperfine parameters, which are centroid shifts, δ, quadrupole shift, ε, magnetic hyperfine field, B_{hf}, magnetic hyperfine field distribution, σ, and intensity, I.

Section S2. Computational study on $CoFe_2O_4$

Computational study was performed by periodic density functional theory (DFT) calculations using QUANTUM ESPRESSO 6.8.[2-4] The obtained data are summarized in the Tables S5–S10 and Figures 7, S2–S5. The DFT+U+J approach[5] was applied to account for the strong correlation among the electrons in the 3d-metal during the geometry optimization of the lattice parameters (atomic positions + cell). All the calculations were carried out with spin polarization and by applying the collinear magnetic model (i.e., all atomic magnetic moments are aligned with the z-axis). The electron−ion interactions for the atoms were described by the projector augmented wave (PAW) method developed by Blöchl.[6] The PAW used in this work were generated by using the atomic package *ld1.x* included in the QUANTUM ESPRESSO distribution (https://www.quantum-espresso.org/documentation/package-specific-documentation). The exchange-correlation energy was calculated within the generalized gradient approximation. PBE (Perdew−Burke−Ernzerhof)[7] and optB86b[8] functionals with and without the semiempirical dispersion corrections developed by Grimme and co-workers (DFT-D3)[9] for the former and van der Waals functional (nonlocal term only) developed by Klimeš and co-authors[10] for the latter were tested by using the ferrimagnetic bulk model of inverse $CoFe_2O_4$. The pseudopotentials were recompiled for optB86b from Andrea Dal Corso's schemes (proposed for PBE and PBEsol) in

pslibrary[11] (https://github.com/dalcorso/pslibrary.git). The optB86b functional with a U_{eff} (U_{eff} = U–J) of 3.6 eV for Fe (U = 4.0 eV and J = 0.4 eV) and 2.5 eV for Co (U = 3.0 eV and J = 0.5 eV) atoms, was then selected to carry out the computational study (see Table S5). U_{eff} follows the Dudarev and co-authors formulation.[12] The kinetic energy cut-offs used in optimization were set to 50 Ry for wavefunction and 500 Ry for charge density and potential. The Monkhorst–Pack scheme was chosen for the integration in the reciprocal space.[13] The reciprocal space of all investigated structures was generally sampled with a 0.20 $Å^{-1}$-spaced *k*-points grid for optimizations and for energy refinements. The projectors for U on localized orbitals were based on Löwdin orthogonalized atomic wavefunctions orbitals ("ortho-atomic" option in QUANTUM ESPRESSO). Marzari–Vanderbilt–Devita–Payne smearing[14] was used for all the calculations. A single point energy refinement of optimized structures was carried out for each most stable entry. The code *projwfc.x* was employed to estimate the atomic magnetic moments and the magnetic moment per formula unit of the bulk and slab collinear models. The computational studies presented in this article are carried out at 0 K and under vacuum conditions.

The crystallographic structure of bulk $CoFe_2O_4$ (COD ID: 1533163) was downloaded from Crystallography Open Database (COD) (http://www.crystallography.net/cod). The bulk and slab computational models of $CoFe_2O_4$ were built based on the cation distribution data obtained from Mössbauer spectroscopy (Table S4) and HAADF–STEM imaging (Figure 5). The slab model was built by cutting the bulk of inverse $CoFe_2O_4$ along the [011] plane of the bulk structure and applying $\begin{bmatrix} 1 & 0 & 0 \\ 0 & 1 & 1 \\ 0 & -1 & 1 \end{bmatrix}$ as rotation matrix. The vacuum gap used was of ca. 13 Å and the stoichiometry of $CoFe_2O_4$ (i.e., 1:2:4) is preserved in the slab model (112 atoms in total).

The space groups are reported in Hermann–Mauguin symmetry symbols. CS and C indicate the Crystallographic System and the cubic symmetry, respectively. The cell parameters are indicated as a;b;c (in Å), α;β;γ (in °) and the cell volume is in $Å^3$. Average M–O bond distances are reported in Å, where *M* is the 3d-transition metal in the octahedral (O_h) or tetrahedral (T_d) site. Magnetic orderings are labelled as: FM (ferromagnetic), AFM (antiferromagnetic), ferri (ferrimagnetic), NM (nonmagnetic, i.e., non-spin polarized calculation). For all the calculations only the estimated spin components of the magnetic moments (μ, in μ_B) are reported. The material type of conduction is indicated with an S, which means the sample is a semiconductor.

Pictures and surfaces are created using VESTA program.[15] Fe, Co, and O are colored in brown, blue, and red, respectively. The isosurfaces in the density spin plots are shown at 0.004 a_0^{-3} (a_0 is the Bohr radius).

Section S3. Conventional electrochemical measurements

The catalyst ink was prepared by ultrasonically dispersing 4 mg of the selected CFO–1173 K nanowires and 1 mg of Vulcan XC 72 carbon black into 1 mL of a solution of ethanol and water at ratio of 3:2 containing 60 µL of Nafion perfluorinated resin solution (Sigma-Aldrich, 5% in mixture of lower aliphatic alcohols and water, contains 45% water) for 1 h (Figure S6a). The

electrode substrate Ni felt (Bekipor 2NI 30-0,50, Bekaert) was loaded drop by drop with the catalyst and let dry under a lamp (Figure S6b). All electrocatalytic tests were carried out in a three-electrode configuration at room temperature using a Biologic VMP-3 potentiostat/galvanostat (Figure S6c). The catalyst supported on Ni felt, a Pt mesh, and a saturated calomel electrode (SCE) were utilized as working, counter, and reference electrodes, respectively (Figure S6d). 1.0 M NaOH was used as electrolyte (Figure S6e). All potentials are reported versus a reversible hydrogen electrode (RHE) by converting the potentials according to the following equation:[16] $E_{\mathrm{RHE}} = E_{\mathrm{SCE}} + 0.059 \times \mathrm{pH} \times 0.241$. Cyclic voltammetry (CV) was performed at scan rate of 5 mV/s in the potential range of 1.0 V_{RHE} to 1.8 V_{RHE}.

Section S4. Magnetoelectrochemical measurements

The electrochemical cell is made of transparent acrylic sheets to a rectangular prism shape, stable for the use of concentrated alkaline solution. Catalyst ink preparation and coating of the electrode was carried out as described in Section S3 above. The dimension of the cell is 4 cm × 3.4 cm × 8 cm, in which 60 mL of 1.0 M NaOH is filled. Then, the catalyst-coated area of the Ni felt is placed in the electrolyte so that the face of the Ni is parallel to one of the sides of the cell. The basic sample holder with tantalum metal contact (redox.me) is used to hold the Ni felt electrode. A 2 cm^2 platinum mesh and Hg/HgO (1.0 M NaOH) are used as the counter electrode and reference electrode, respectively. To apply a uniform magnetic field, a dipole electromagnet is used (Figure 8).

The electrochemical measurements were carried out in the following sequence: uncompensated resistance measurement, impedance at open circuit potential, CV at 100 mV/s (50 cycles), CV at 50 mV/s (10 cycles), CV at 10 mV/s (5 cycles), CV at 5 mV/s (5 cycles), chronoamperometry at 0.8$V_{Hg/HgO}$ and linear sweep voltammetry (LSV) at 5 mV/s (5 times). Immediately after completing this sequence, magnetic field is applied: the distance between the poles is 3.5 cm, the power supplied is 5.6 A, and the measured magnetic field between the poles is 500 mT. After 2 min, LSV at 5 mV/s (5 times) was measured. If the current density increased compared to the previous measurement without magnetic field, LSV was continued for a few more cycles to reach saturation in the current density. In case the current density did not increase or if the current density decreased with the magnetic field, the direction of magnetic field was changed. Unfortunately, it is not possible to change the direction of the magnetic field in the electromagnet used in the setup. Therefore, the working electrode is turned 180° and the measurements with the magnetic field continued.

After the initial LSV at 5 mV/s (5 times) under external magnetic field recorded, chronoamperometry at 0.8 $V_{Hg/HgO}$ for 1 h was carried out. After 1 h, the electromagnet was switched off and chronoamperometry continued for a further 9 h.

Monitoring and control of the temperature of the electrolyte during magnetoelectrochemical measurements is important, since prolonged use of the electromagnet will cause an increase in the temperature of the cell. Hence, the electrochemical cell was kept inside the water bath with water circulation to maintain a constant temperature.

TABLES:

Table S1. Powder X-ray diffraction (PXRD) data with Bragg angle, the full width at half maximum (FWHM), and crystallite size D_{PXRD} of $CoFe_2O_4$ materials produced through calcination at different temperatures. The values employed for Figure 1b in the main manuscript are indicated with gray background.

Sample	2θ (°)	FWHM (°)	D_{PXRD} (nm)
**CFO 1273 K**	30.09	0.1688	48.73
	35.45	0.1694	49.24
	37.08	0.1532	54.69
	43.09	0.1549	55.14
	53.49	0.1514	58.75
	57.01	0.1861	48.57
	62.62	0.1658	56.10
	74.10	0.1833	54.30
**CFO 1173 K**	30.08	0.2092	39.32
	35.44	0.2106	39.60
	37.06	0.1427	58.72
	43.08	0.2035	41.96
	53.47	0.1704	52.21
	56.99	0.2297	39.36
	62.59	0.2201	42.24
	74.06	0.2129	46.74
**CFO 1073 K**	30.07	0.3034	27.12
	35.44	0.3380	24.68
	37.06	0.2955	28.35
	43.07	0.2958	28.88
	53.44	0.2894	30.74
	56.99	0.4465	20.24
	62.58	0.3641	25.53
	74.03	0.2994	33.24
**CFO 973 K**	30.09	0.3757	21.89
	35.45	0.4033	20.68
	37.07	0.3378	24.81
	43.10	0.3687	23.16
	53.48	0.3403	26.14
	57.00	0.4409	20.50
	62.59	0.4068	22.86
	74.06	0.3010	33.07
**CFO 873 K**	30.09	0.5410	15.21
	35.43	0.5842	14.28
	37.04	0.3122	26.84
	43.07	0.5585	15.29
	53.52	1.3731	6.48
	62.58	0.5329	17.44

Table S2. The PXRD intensity ratios I_{220}/I_{222}, I_{422}/I_{222} and the crystallite size for $CoFe_2O_4$ produced through calcination at temperatures of 873 K, 973 K, 1073 K, 1173 K, and 1273 K.

Calcination temperature (K)	I_{220}/I_{222}	I_{422}/I_{222}	Crystallite size (nm)
873	2.74	1.58	13.74
973	2.32	1.29	21.82
1073	1.75	0.97	25.29
1173	3.50	1.40	40.60
1273	3.58	1.41	51.56

Table S3. Coercivity H_c, remanent magnetization M_r, and saturation magnetization M_s measured at 2 K and 300 K for the $CoFe_2O_4$ nanomaterials produced through calcination at different temperatures.

**Calcination *T*** **K**	**Measurement *T*** **K**	H_c **Oe**	M_r **emu/g**	M_s[a] **emu/g**
873	2	16400	69.6	81.9
	300	635	24.5	88.1
973	2	15900	62.2	76.6
	300	943	18.4	75.0
1073	2	16300	57.7	70.1
	300	1175	24.1	71.3
1173	2	17100	76.6	88.9
	300	1736	38.6	92.6
1273	2	16600	72.9	81.9
	300	2500	39.3	87.3

[a] Estimated by extrapolating M vs $1/H$ for $1/H \rightarrow 0$.

Table S4. Summary of refined Mössbauer parameters for CFO–1173 K for both spectra as indicated in the table, measured at 6 K and 293 K: centroid shift, δ, quadrupole shift, ε, magnetic hyperfine field, B_{hf}, magnetic hyperfine field distribution, σ, and intensity, I. Estimated errors are in I ±3%, in δ and ε ±0.005 mm/s, and in B_{hf} and σ ±0.2 T.

		CFO–1173 K	
	T (K)	6	293
	δ_1 (mm/s)	0.462	0.354
	B_{hf1} (T)	55.1	52.3
	σ_1 (T)	0.7	0.6
Q_1	ε_1 (mm/s)	0.004	−0.012
	I_1 (%)	36	30
	δ_2 (mm/s)	0.545	0.439
	B_{hf2} (T)	53.7	49.8
	σ_2 (T)	1.4	0.8
Q_2	ε_2 (mm/s)	−0.047	0.008
	I_2 (%)	15	20
	δ_3 (mm/s)	0.349	0.208
	B_{hf3} (T)	51.3	49.2
	σ_3 (T)	0.7	0.7
Q_3	ε_3 (mm/s)	−0.005	−0.016
	I_3 (%)	49	50

Table S5. Experimental data on bulk $CoFe_2O_4$.[17-20]

**Space group**	**CS**	**Cell parameters (Å)**	**Cell volume (Å^3)**	**Average M-O distance (Å)**	**Magn. Struc. type**	***T*$_c$ (K)**	**Mag. Mom. μ_B**	**Cond. type**
Fd-3m:2	C	a=b=c= 8.3806; α=β=γ=90º	588.607	2.04	ferri	790 (inverse spinel)	3	S

Table S6. Lattice parameters (in Å), cell volume (in $Å^3$), estimated average *M*–O distance (in Å), estimated absolute average magnetic moments (spin-only component) (in μ_B) and minimum band gaps (in eV) of ferrimagnetic $CoFe_2O_4$ bulk inverse spinel calculated with various exchange-correlation functionals. Available experimental data are reported in Table S5 and in this table for comparison. The chosen computational method for this work **is highlighted in bold**. N.R. indicates that the data have not been reported and/or are not available in literature.

Methods	Cell parameters (Å)	Cell volume ($Å^3$)	Average estimated M-O distance (Å)	Abs. Average Mag. Mom. ($\|\mu_B\|$)	Minimum band gap (eV)
Experiment	a=b=c=8.3806; α=β=γ=90°	588.607	2.04	N.R.	1.2 (indirect E_g) and 2.7 (direct E_g) (thin films); [21] 0.11-2.6 [21]
PBE+U+J (U_{Fe}=4.5 and J_{Fe}=0.4 eV; U_{Co}=3.0 and J_{Co}=0.5 eV)	a=8.35367, b=8.35016, c=8.36301; α=89.86°, β=90.10°, γ=90.23°	610.92	1.92 (Fe-O T_d) 2.06 (Fe-O O_h) 2.10 (Co-O O_h)	4.1 (Fe) 2.6 (Co)	1.79 (spin ↑) 1.32 (spin ↓)
PBE+U+J+D3M (U_{Fe}=4.5 and J_{Fe}=0.4 eV; U_{Co}=3.0 and J_{Co}=0.5 eV)	a=8.35367, b=8.35016, c=8.36301; α=89.86°, β=90.10°, γ=90.24°	583.35	1.89 (Fe-O T_d) 2.03 (Fe-O O_h) 2.06 (Co-O O_h)	4.1 (Fe) 2.6 (Co)	1.82 (spin ↑) 1.26 (spin ↓)
optB86b+U+J (U_{Fe}=4.5 and J_{Fe}=0.4 eV; U_{Co}=3.0 and J_{Co}=0.5 eV)	a=8.38473, b=8.38090, c=8.39342; α=89.88°, β=90.08°, γ=90.22°	589.81	1.90 (Fe-O T_d) 2.04 (Fe-O O_h) 2.07 (Co-O O_h)	4.1 (Fe) 2.6 (Co)	1.78 (spin ↑) 1.14 (spin ↓)
optB86bvdw+U+J (U_{Fe}=4.5 and J_{Fe}=0.4 eV; U_{Co}=3.0 and J_{Co}=0.5 eV)	a=8.42140, b=8.41705, c=8.43081; α=89.88°, β=90.08°, γ=90.21°	597.60	1.91 (Fe-O T_d) 2.05 (Fe-O O_h) 2.08 (Co-O O_h)	4.1 (Fe) 2.6 (Co)	1.74 (spin ↑) 1.20 (spin ↓)
**optB86b+U+J (U_{Fe}=4.0 and J_{Fe}=0.4 eV; U_{Co}=3.0 and J_{Co}=0.5 eV)**	**a=8.37836, b=8.37387, c=8.38739; α=89.89°, β=90.07°, γ=90.20°**	**588.45**	**1.90 (Fe-O T_d) 2.04 (Fe-O O_h) 2.06 (Co-O O_h)**	**4.0 (Fe) 2.6 (Co)**	**1.73 (spin ↑) 1.06 (spin ↓)**

Table S7. Optimized computational data on inverse $CoFe_2O_4$ bulk (T = 0 K) obtained by using the collinear magnetic model with DFT+U+J (optB86b, U_{Fe}=4.0 and J_{Fe}=0.4 eV, U_{Co}=3.0 and J_{Co}=0.5 eV) method. Lattice parameters (in Å), cell volume (in $Å^3$), estimated average *M*–O distance (in Å), and estimated absolute average magnetic moments (spin-only component) (in μ_B) are reported. The most stable ground state is referred to as 0.00 eV.

**CS (C)**	**Magn. Struc. type**	**ΔE (eV)**	**Cell parameters (Å)**	**Cell volume ($Å^3$)**	**Average M-O distance (Å)**	**Abs. Average Mag. Mom. ($\|\mu_B\|$)**
**Fd-3m**	NM	44.58	a=8.15133; b=8.09743; c=7.62021; α=89.97º; β=89.86º; γ=91.50º	502.80	1.85 (Fe-O T_d) 1.95 (Fe-O O_h) 1.91 (Co-O O_h)	-
	FM	No convergence				
	AFM	No convergence				
	**Ferri1**	**0.00**	**a=8.37836; b=8.37387; c=8.38739; α=89.89º; β=90.07º; γ=90.20º**	**588.45**	**1.90 (Fe-O T_d) 2.04 (Fe-O O_h) 2.06 (Co-O O_h)**	**4.0 (Fe) 2.6 (Co)**
	Ferri2	2.29	a=8.40522; b=8.39665; c=8.42469; α=89.77º; β=90.13º; γ=90.23º	594.57	1.91 (Fe-O T_d) 2.03 (Fe-O O_h) 2.08 (Co-O O_h)	4.1 (Fe) 2.7 (Co)

Table S8. Computational data on ferrimagnetic (011) CFO–1173 K (T = 0 K).

**Cell parameters (Å)**	**Cell volume (Å^3)**
a=8.41880; b=11.95685; c=25.10454;<br>α=90.31º; β=90.36º; γ=90.42º	2526.93

Table S9. Experimental magnetic data on CFO–1173 K nanowires (see also Table S3) and on bulk.[22] The magnetic moments per formula unit (f. u.) in Bohr magneton (μ_B) of CFO–1173 K are obtaining considering the molar mass of $CoFe_2O_4$ (M =234.621 g/mol), the Bohr magneton in SI unit (0.92731×10^{-23} A m^2/molecule) and the Avogadro's constant (N = $6.0249 \cdot 10^{23} \times$ atoms/mol).[23]

**Measured T (K)**	**Saturation magnetization (M_s) (emu/g)**	**Saturation magnetization (M_s) ($A \cdot m^2/Kg$)**	**Saturation magnetization (M_s) ($A \cdot m^2/g$)**	**Magnetic moment per formula unit (f. u.) ($A \cdot m^2/mol$)**	**Magnetic moment per formula unit (f. u.) (μ_B)**
2	88.9	88.9	0.0889	20.86	3.74
300	92.6	92.6	0.0926	21.73	3.89
bulk	80.8	80.8	0.0808	18.96	3.39

Table S10. Comparison between computational and experimental magnetic moment per formula unit (f. u.) of CFO–1173 K nanowires and bulk CFO. Computational magnetic moments (spin-only component) per f. u. are estimated with *projwfc.x* code.

	**Exp. CFO–1173 K at 2K**	**Comp. (011) CFO–1173 K slab model (spin-only component)**	**Exp. Bulk CFO**	**Comp. bulk CFO (spin-only component)**
**Magnetic moment per formula unit (f. u.) (μ_B)**	3.74	3.34	3.39	3.04

Table S11. Performance of CFO–1173 K in OER.

Magnetic field *H*	η_{10}	η_{20}	η_{50}	η_{100}	Tafel slope (mV)	Magnetocurrent (from chronoamperometry) (mA/cm^2)	% of enhancement
	(mV)						
**0 mT**	318	341	386	448	50	–	
**500 mT**	292	307	330	353	43	51.7	100

Table S12. Performance of pristine Ni felt and commercial benchmarks in alkaline OER.

**Catalyst**	**Magnetic field**	η_{10} **mV**	η_{100} **mV**	**Tafel slope (mV/dec)**	**Magnetocurrent (from LSV) at 1.6 V_{RHE} (mA cm^{-2})**	**% of enhancement**
Ni felt (Bekaert)	0 mT	313	430	93	–	
	500 mT	300	387	69	34	83
IrO_2 (TKK, TEC77100)	0 mT	186	275	76	–	
	500 mT	183	275	76	0	0
$NiFe_2O_4$ (US Research Nanomaterials)	0 mT	277	343	55	–	
	500 mT	256	327	56	27	15

Table S13. Comparison of the increase in performance of CFO–1173 K under external magnetic field as compared to literature-known catalysts.

Catalyst material	Magnetocurrent (%)	E (V) vs RHE	Magnetic Field (T)	Reference
$CoFe_2O_4$	≈0.8	1.66	1	[24]
$CoFe_2O_4$	≈200%	1.64	nD	[25]
$NiFe_2O_4$	≈20%	1.70	1	[26]
$SmCo_5$/CoOxH	110%	1.65	1.25	[27]
NiCoFe-LDH	≈35%	nD	0.7	[28]
LSCMO	75%	1.80	0.5	[29]
CFO–1173 K	100%	1.60	0.5	This work

FIGURES:

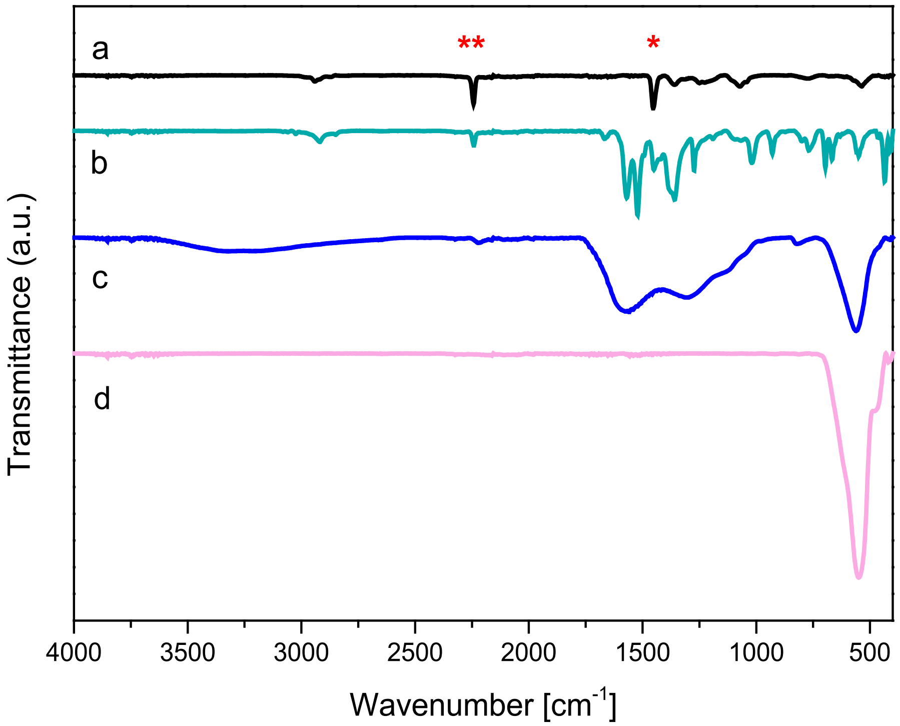

Figure S1. FTIR spectra of (a) as-received polyacrylonitrile (PAN) polymer powder, (b) Co- and Fe-containing as-spun PAN fibers, (c) after thermal stabilization at 573 K for 2 h in air, and (d) after calcination at 1173 K for 2 h in air. **Characteristic absorption peaks of nitrile (–C≡N) group at around 2240 cm^{-1}; *aliphatic CH groups.[30]

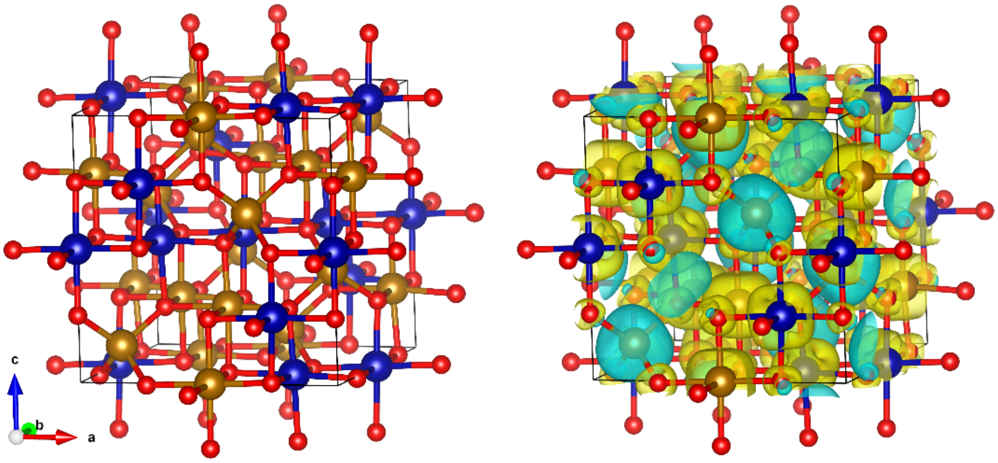

Figure S2. Optimized cell (right) and spin density plot (left) of ferrimagnetic (Ferri 1) bulk $CoFe_2O_4$ inverse spinel (yellow = spin up, cyan = spin down).

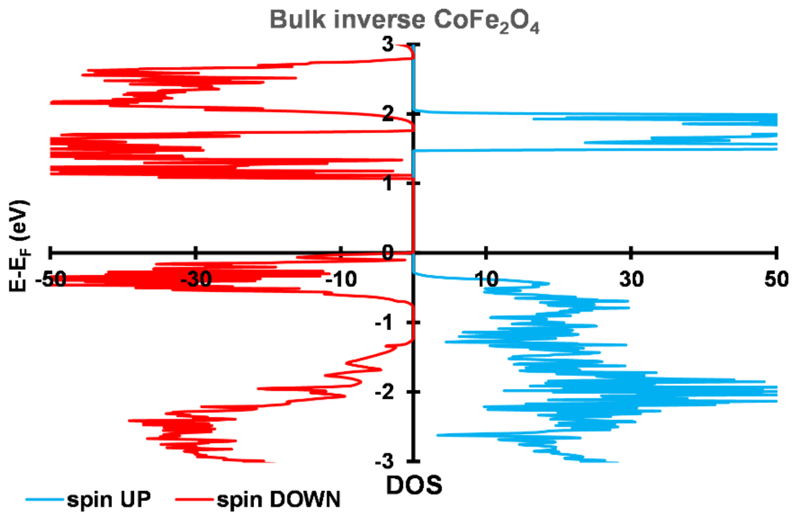

Figure S3. Density of states (DOS) between –3 eV and 3 eV of ferrimagnetic (Ferri 1) bulk $CoFe_2O_4$ inverse spinel (cyan = spin up, red = spin down).

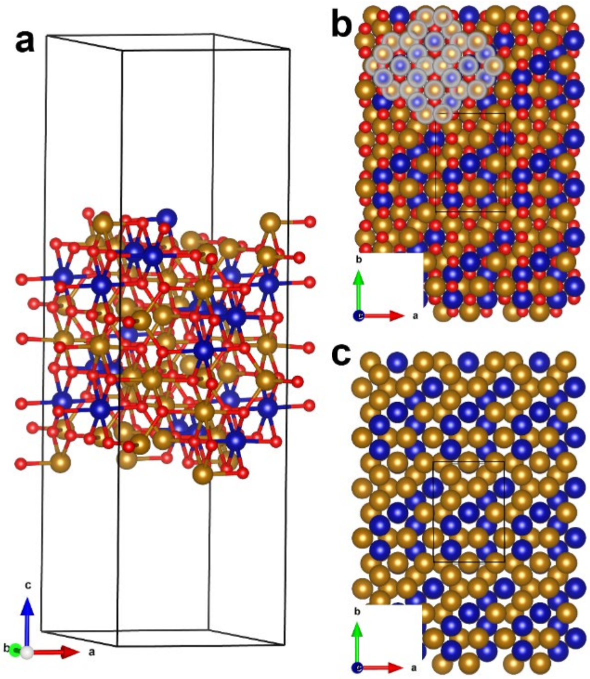

Figure S4. (a) Slab model of (011) inverse $CoFe_2O_4$ spinel (CFO–1173 K) used in this work. (b) and (c) Top views of the (011) $CoFe_2O_4$ surface with all the atoms and without oxygen atoms, respectively.

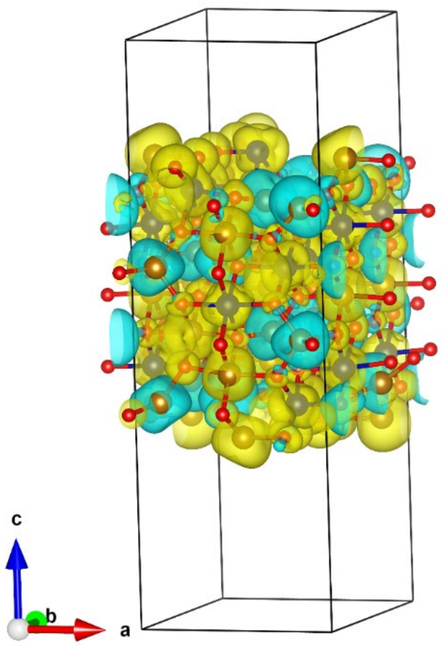

Figure S5. Spin density plot of ferrimagnetic (011) slab model of inverse $CoFe_2O_4$ spinel (yellow = spin up, cyan = spin down).

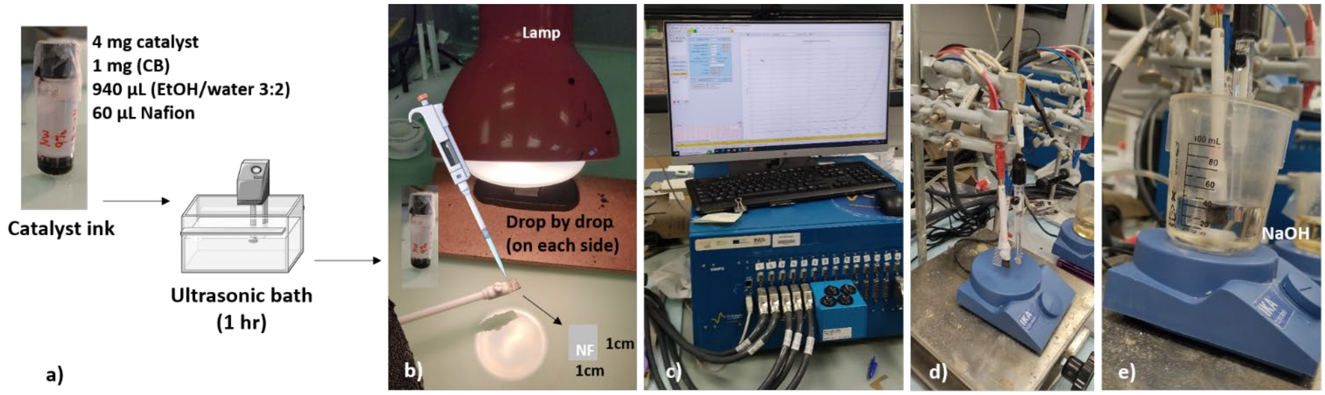

Figure S6. (a–e) Workflow of conventional electrochemical study.

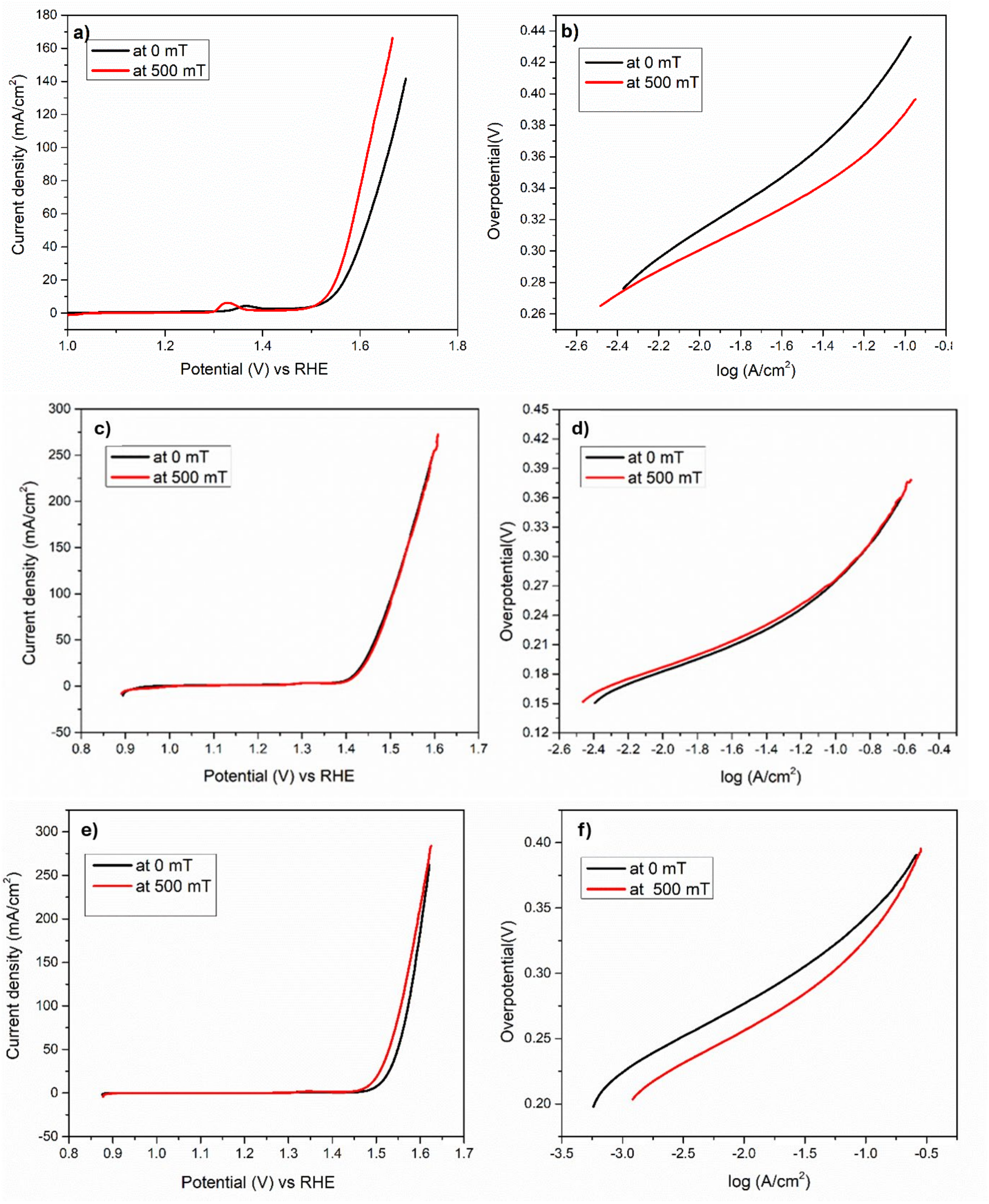

Figure S7. OER anodic polarization curves and Tafel plots of (a,b) control pristine Ni felt (Bekaert), as well as reference (c,d) IrO_2 (TKK, TEC77100) and (e,f) $NiFe_2O_4$ (20 nm, US Research Nanomaterials) benchmark catalysts in the absence and presence of an external magnetic field of 500 mT, all recorded in 1.0 M NaOH at room temperature. All polarization curves are 85% *iR*-compensated.

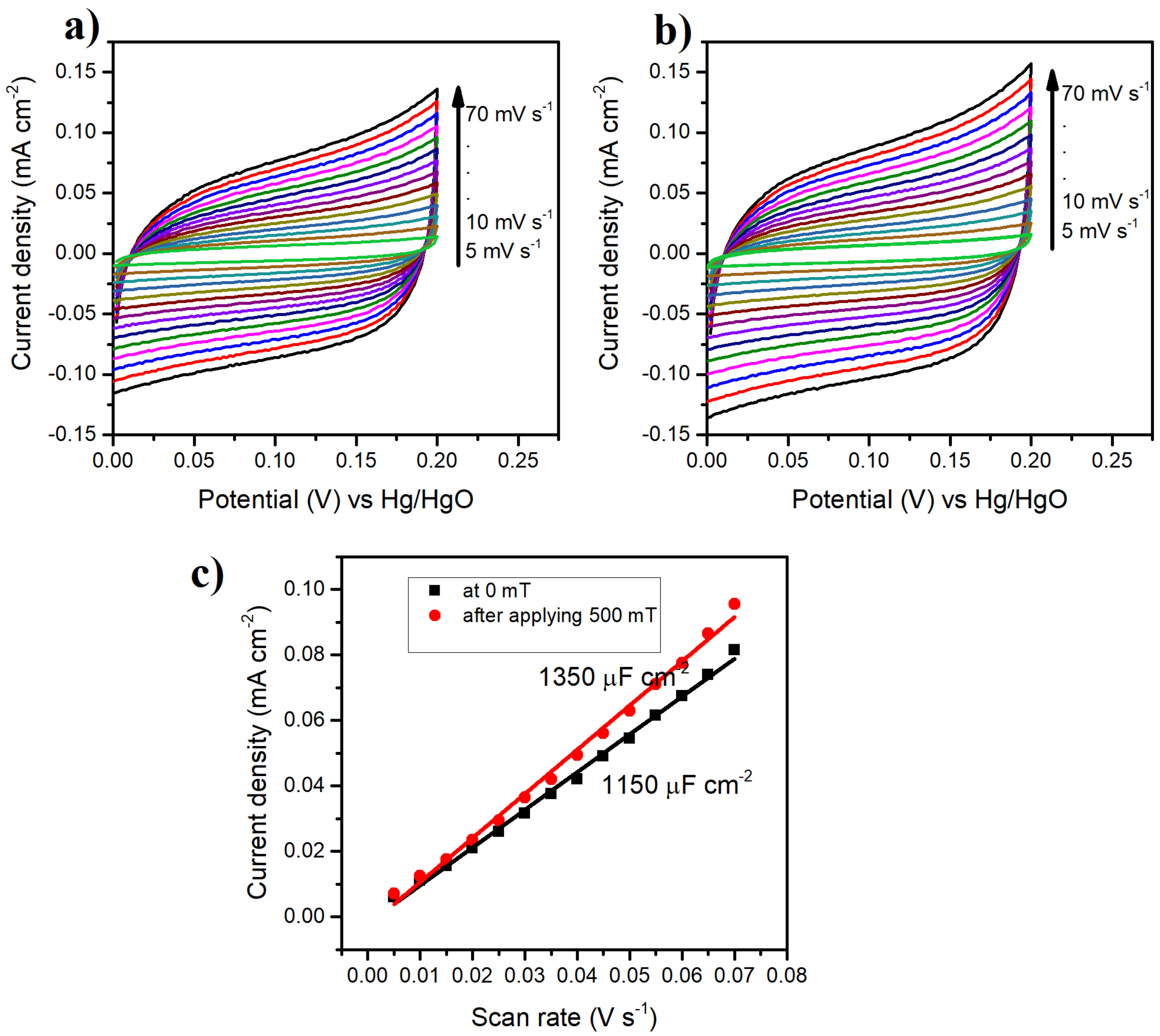

Figure S8. Cyclic voltammetry scans performed at scan rates of 5 to 70 mV/s (a) in the absence of magnetic field and (b) with the applied magnetic field of 500 mT, together with (c) geometric double-layer capacitance plots for CFO–1173 K.

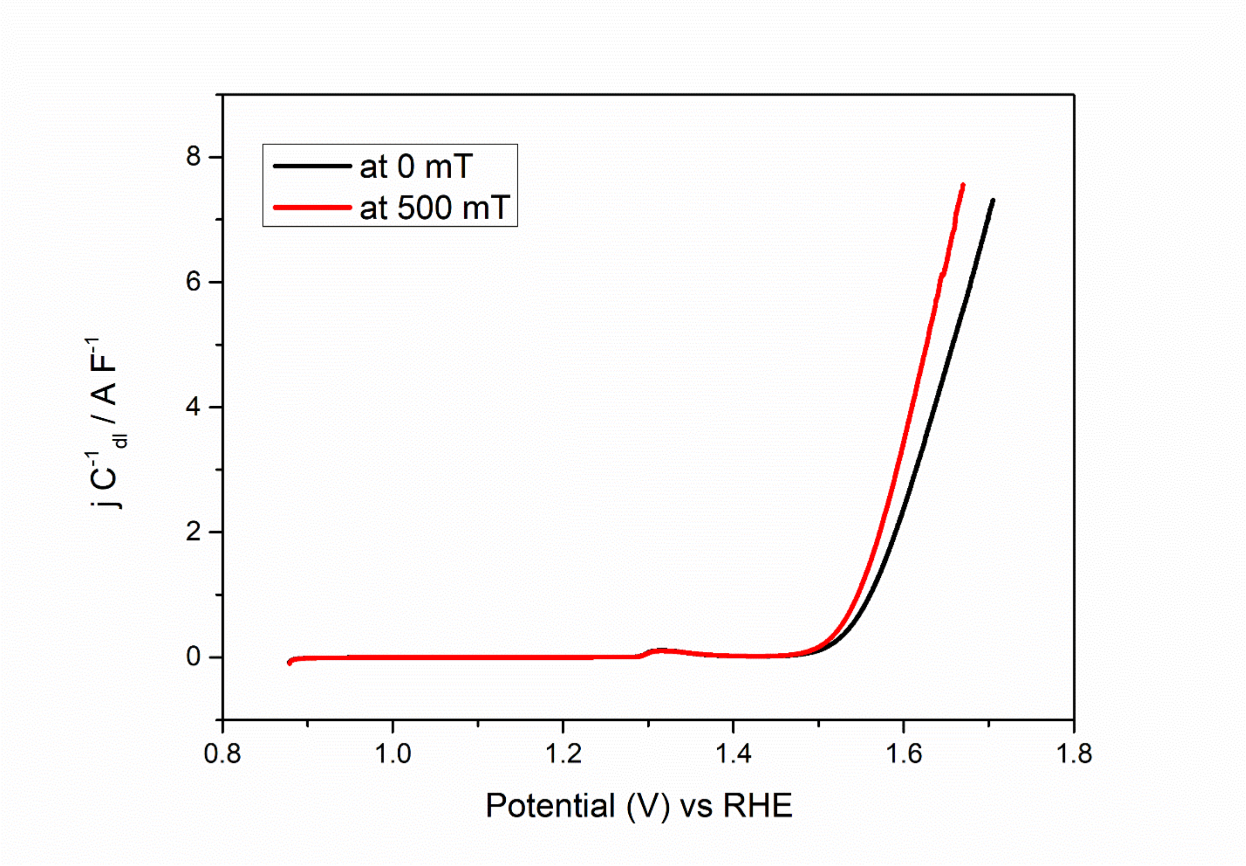

Figure S9. Cyclic voltammetry data of CFO–1173 K presented in Figure 9a, with currents normalized to double layer capacitance calculated in Figure S9c.

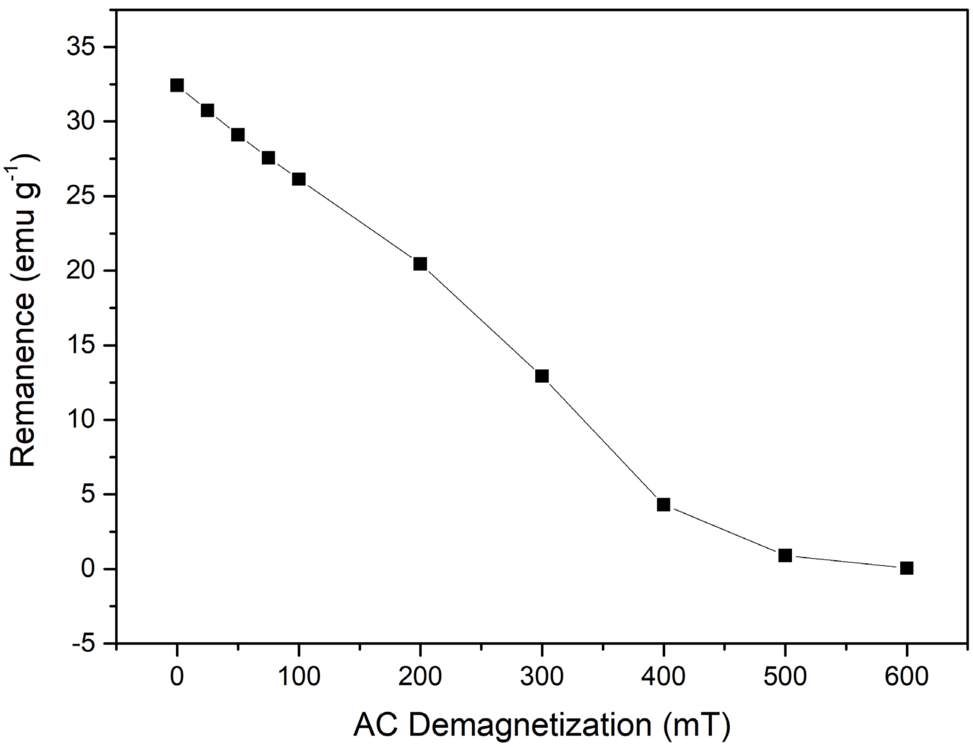

Figure S10. Demagnetization of CFO−1173 K.

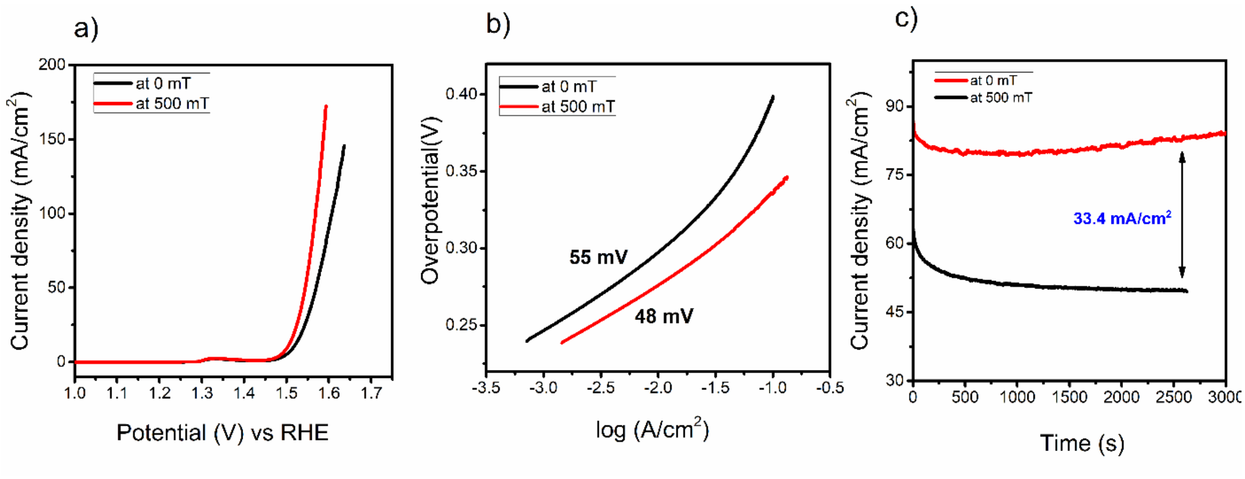

Figure S11. Magnetoelectrochemical data of CFO–1273 K with (H = 500 mT, red) and without (H = 0 mT, black) an external magnetic field: (a) LSV at the scan rate of 5 mV/s, (b) Tafel plots extracted from the collected LSV data, (c) chronoamperometry curves during 1 h at 1.67 V_{RHE}.

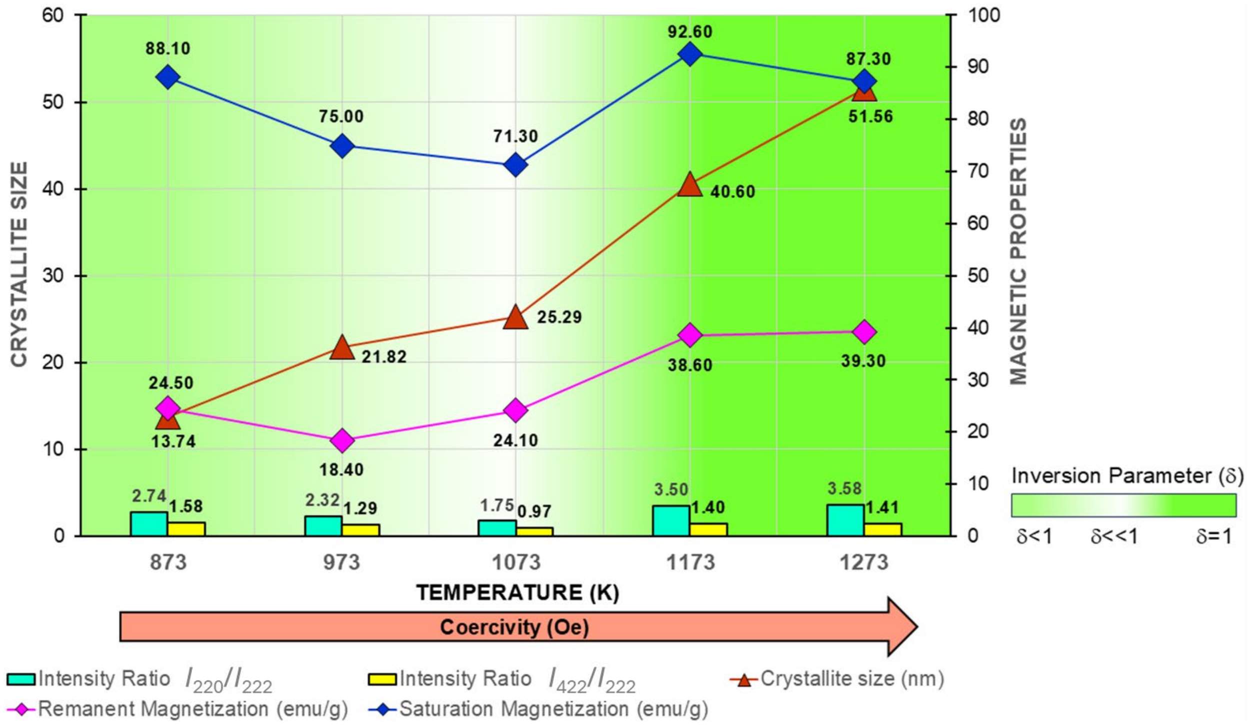

Figure S12. Physical and magnetic properties of CFO with respect to the calcination temperature.

References

1. Lagarec, K., *Recoil User Manual -- Mössbauer spectral analysis software for Windows*. 1998.

2. Giannozzi, P.; Andreussi, O.; Brumme, T.; Bunau, O.; Buongiorno Nardelli, M.; Calandra, M.; Car, R.; Cavazzoni, C.; Ceresoli, D.; Cococcioni, M.; Colonna, N.; Carnimeo, I.; Dal Corso, A.; de Gironcoli, S.; Delugas, P.; DiStasio, R. A.; Ferretti, A.; Floris, A.; Fratesi, G.; Fugallo, G.; Gebauer, R.; Gerstmann, U.; Giustino, F.; Gorni, T.; Jia, J.; Kawamura, M.; Ko, H. Y.; Kokalj, A.; Küçükbenli, E.; Lazzeri, M.; Marsili, M.; Marzari, N.; Mauri, F.; Nguyen, N. L.; Nguyen, H. V.; Otero-de-la-Roza, A.; Paulatto, L.; Poncé, S.; Rocca, D.; Sabatini, R.; Santra, B.; Schlipf, M.; Seitsonen, A. P.; Smogunov, A.; Timrov, I.; Thonhauser, T.; Umari, P.; Vast, N.; Wu, X.; Baroni, S., Advanced capabilities for materials modelling with Quantum ESPRESSO. *Journal of Physics C* **2017,** *29*, 465901.

3. Giannozzi, P.; Baroni, S.; Bonini, N.; Calandra, M.; Car, R.; Cavazzoni, C.; Ceresoli, D.; Chiarotti, G. L.; Cococcioni, M.; Dabo, I.; Dal Corso, A.; de Gironcoli, S.; Fabris, S.; Fratesi, G.; Gebauer, R.; Gerstmann, U.; Gougoussis, C.; Kokalj, A.; Lazzeri, M.; Martin-Samos, L.; Marzari, N.; Mauri, F.; Mazzarello, R.; Paolini, S.; Pasquarello, A.; Paulatto, L.; Sbraccia, C.; Scandolo, S.; Sclauzero, G.; Seitsonen, A. P.; Smogunov, A.; Umari, P.; Wentzcovitch, R. M., QUANTUM ESPRESSO: a modular and open-source software project for quantum simulations of materials. *Journal of Physics C* **2009,** *21*, 395502.

4. Giannozzi, P.; Baseggio, O.; Bonfà, P.; Brunato, D.; Car, R.; Carnimeo, I.; Cavazzoni, C.; de Gironcoli, S.; Delugas, P.; Ferrari Ruffino, F.; Ferretti, A.; Marzari, N.; Timrov, I.; Urru, A.; Baroni, S., Quantum ESPRESSO toward the exascale. *Journal of Physics C* **2020,** *152*, 154105.

5. Cococcioni, M.; de Gironcoli, S., Linear response approach to the calculation of the effective interaction parameters in the LDA + U method. *Phys. Rev. B* **2005,** *71*, 035105.

6. Blöchl, P. E., Projector augmented-wave method. *Phys. Rev. B* **1994,** *50*, 17953—17979.

7. Perdew, J. P.; Burke, K.; Ernzerhof, M., Generalized Gradient Approximation Made Simple. *Phys. Rev. Lett.* **1996,** *77*, 3865—3868.

8. Klimeš, J.; Bowler, D. R.; Michaelides, A., Chemical accuracy for the van der Waals density functional. *Journal of Physics C* **2010,** *22*, 022201.

9. Grimme, S.; Antony, J.; Ehrlich, S.; Krieg, H., A consistent and accurate ab initio parametrization of density functional dispersion correction (DFT-D) for the 94 elements H-Pu. *J. Chem. Phys* **2010,** *132*.

10. Klimeš, J.; Bowler, D. R.; Michaelides, A., Van der Waals density functionals applied to solids. *Phys. Rev. B* **2011,** *83*, 195131.

11. Dal Corso, A., Pseudopotentials periodic table: From H to Pu. *Comput. Mater. Sci.* **2014,** *95*, 337—350.

12. Dudarev, S. L.; Botton, G. A.; Savrasov, S. Y.; Humphreys, C. J.; Sutton, A. P., Electron-energy-loss spectra and the structural stability of nickel oxide: An LSDA+U study. *Phys. Rev. B* **1998,** *57*, 1505—1509.

13. Monkhorst, H. J.; Pack, J. D., Special points for Brillouin-zone integrations. *Phys. Rev. B* **1976,** *13*, 5188—5192.
14. Marzari, N.; Vanderbilt, D.; De Vita, A.; Payne, M. C., Thermal Contraction and Disordering of the Al(110) Surface. *Phys. Rev. Lett.* **1999,** *82*, 3296—3299.
15. Momma, K.; Izumi, F. J. J. o. A. C., VESTA 3 for three-dimensional visualization of crystal, volumetric and morphology data. **2011,** *44*, 1272—1276.
16. Amorim, I.; Yu, Z.; Bento, F.; Liu, L., Towards an Improved Electrocatalytic Material for Detection of Polyphenols Based on Transition Metal Phosphides Anchored on Reduced Graphene Oxide. *J. Electrochem. Soc.* **2023,** *170*, 027506.
17. Arteaga-Cardona, F.; Pal, U.; María Alonso, J.; de la Presa, P.; Mendoza-Álvarez, M.-E.; Salazar-Kuri, U.; Méndez-Rojas, M. Á., Tuning magnetic and structural properties of $MnFe_2O_4$ nanostructures by systematic introduction of transition metal ions M^{2+} (M = Zn, Fe, Ni, Co). *J. Magn. Magn. Mater.***2019,** *490*, 165496.
18. Coey, J. M. D., *Magnetism and Magnetic Materials*. Cambridge University Press: Cambridge, 2010.
19. Ferreira, T. A. S.; Waerenborgh, J. C.; Mendonça, M. H. R. M.; Nunes, M. R.; Costa, F. M., Structural and morphological characterization of $FeCo_2O_4$ and $CoFe_2O_4$ spinels prepared by a coprecipitation method. *Solid State Sci.***2003,** *5*, 383—392.
20. Panda, R. K.; Behera, D., Investigation of electric transport behavior of bulk $CoFe_2O_4$ by complex impedance spectroscopy. *J. Alloys Compd.* **2014,** *587*, 481—486.
21. Holinsworth, B. S.; Mazumdar, D.; Sims, H.; Sun, Q.-C.; Yurtisigi, M. K.; Sarker, S. K.; Gupta, A.; Butler, W. H.; Musfeldt, J. L., Chemical tuning of the optical band gap in spinel ferrites: $CoFe_2O_4$ vs $NiFe_2O_4$. *Applied Physics Letters* **2013,** *103*.
22. Gopalan, E. V.; Joy, P. A.; Al-Omari, I. A.; Kumar, D. S.; Yoshida, Y.; Anantharaman, M. R., On the structural, magnetic and electrical properties of sol–gel derived nanosized cobalt ferrite. *J. Alloys Compd.* **2009,** *485*, 711—717.
23. Mabbs, F. E.; Machin, D. J., *Magnetism and Transition Metal Complexes*. Chapman and Hall: 1973.
24. Wu, T.; Ren, X.; Sun, Y.; Sun, S.; Xian, G.; Scherer, G. G.; Fisher, A. C.; Mandler, D.; Ager, J. W.; Grimaud, A.; Wang, J.; Shen, C.; Yang, H.; Gracia, J.; Gao, H.-J.; Xu, Z. J., Spin pinning effect to reconstructed oxyhydroxide layer on ferromagnetic oxides for enhanced water oxidation. *Nat. Commun.* **2021,** *12*, 3634.
25. Ge, J.; Ren, X.; Chen, R. R.; Sun, Y.; Wu, T.; Ong, S. J. H.; Xu, Z. J., Multi-Domain versus Single-Domain: A Magnetic Field is Not a Must for Promoting Spin-Polarized Water Oxidation. *Angew. Chem. Int. Ed.* **2023,** *62*, e202301721.
26. Lyu, X.; Zhang, Y.; Du, Z.; Chen, H.; Li, S.; Rykov, A. I.; Cheng, C.; Zhang, W.; Chang, L.; Kai, W.; Wang, J.; Zhang, L.; Wang, Q.; Huang, C.; Kan, E., Magnetic Field Manipulation of Tetrahedral Units in Spinel Oxides for Boosting Water Oxidation. *Small* **2022,** *18*, 2204143.

27. Chen, R. R.; Chen, G.; Ren, X.; Ge, J.; Ong, S. J. H.; Xi, S.; Wang, X.; Xu, Z. J., $SmCo_5$ with a Reconstructed Oxyhydroxide Surface for Spin-Selective Water Oxidation at Elevated Temperature. *Angew. Chem. Int. Ed.* **2021,** *60*, 25884—25890.
28. Lin, L.; Xin, R.; Yuan, M.; Wang, T.; Li, J.; Xu, Y.; Xu, X.; Li, M.; Du, Y.; Wang, J.; Wang, S.; Jiang, F.; Wu, W.; Lu, C.; Huang, B.; Sun, Z.; Liu, J.; He, J.; Sun, G., Revealing Spin Magnetic Effect of Iron-Group Layered Double Hydroxides with Enhanced Oxygen Catalysis. *ACS Catal.* **2023,** *13*, 1431—1440.
29. Xu, H.; Qi, J.; Zhang, Y.; Liu, H.; Hu, L.; Feng, M.; Lü, W., Magnetic Field-Enhanced Oxygen Evolution Reaction via the Tuneability of Spin Polarization in a Half-Metal Catalyst. *ACS Appl. Mater. Interfaces* **2023,** *15*, 32320—32328.
30. Li, J.; Su, S.; Zhou, L.; Kundrát, V.; Abbot, A. M.; Mushtaq, F.; Ouyang, D.; James, D.; Roberts, D.; Ye, H., Carbon nanowalls grown by microwave plasma enhanced chemical vapor deposition during the carbonization of polyacrylonitrile fibers. *J. Appl. Phys* **2013,** *113*.
